# Supplementary material for: High-throughput laboratory evolution reveals evolutionary constraints in Escherichia coli
Source: Nat Commun. 2020 Nov 24;11:5970. doi: 10.1038/s41467-020-19713-w (PMC7686311; doi:10.1038/s41467-020-19713-w)
Supplement: Supplementary file 1 — Supplementary Information [file 41467_2020_19713_MOESM1_ESM.pdf]

Supplementary Information for
High-throughput laboratory evolution reveals evolutionary constraints in *Escherichia coli*

Tomoya Maeda[1,&*], Junichiro Iwasawa[2,&], Hazuki Kotani[1], Natsue Sakata[1], Masako Kawada[1], Takaaki Horinouchi[1], Aki Sakai[1], Kumi Tanabe[1], Chikara Furusawa[1,2,3*]

[1]RIKEN Center for Biosystems Dynamics Research, 6-2-3 Furuedai, Suita, Osaka 565-0874, Japan
[2]Department of Physics, The University of Tokyo, 7-3-1 Hongo, Tokyo 113-0033, Japan
[3]Universal Biology Institute, The University of Tokyo, 7-3-1 Hongo, Tokyo 113-0033, Japan

[&]These authors contributed equally to this work.

Supplementary Discussion: Detailed description of common mutations identified in evolved strains.

Each laboratory evolution condition was carried out with six independent culture lines. Four evolved strains isolated from different culture lines were used for further phenotypic, transcriptomic, and genotypic analyses. Common mutations were identified in these four evolved strains.

3-AT evolved strains

It was reported that 3-AT inhibits histidine biosynthesis [1]. Since purine and L-histidine biosynthesis are linked by imidazole glycerol phosphate synthetase encoded by *hisHF* genes, the addition of adenine relieves the growth inhibition by 3-AT in *E. coli* [1]. Two 3-AT evolved strains had mutations in the *apt* locus encoding adenine phosphoribosyltransferase (Supplementary Data 4). A reconstructed *apt* inactivation mutant strain also showed a 3-fold increase in 3-AT resistance (Supplementary Data 2). Inactivation of adenine phosphoribosyltransferase which converts adenine to AMP can result in an increase in adenine pool and 3-AT resistance.

5-FOA evolved strains

5-FOA is a nucleobase analog which is converted into the toxic compound 5-fluorouracil by orotine-5′-monophosphate decarboxylase (OMP) encoded by *pyr*F in *E. coli* [2]. Three 5-FOA evolved strains had mutations in *dctA* encoding C4‑dicarboxylic acid, orotate, and citrate transporter (Supplementary Data 4). A reconstructed *dctA* inactivation mutant strain also showed a 30-fold increase in 5-FOA resistance (Supplementary Data 2). These results strongly suggest the uptake of 5-FOA by the transporter.

5-FU evolved strains

5-FU is an uracil analog that inhibits DNA synthesis by mainly inhibiting thymidylate synthase. Three 5-FU evolved strains had mutations in *uraA* encoding uracil: H^+ symporter UraA (Supplementary Data 4). A reconstructed *uraA* inactivation mutant strain also showed an 8-fold increase in 5-FU resistance (Supplementary Data 2). These results strongly suggest the uptake of 5-FU by the transporter.

6-MP evolved strains

6-MP is a purine analogue which inhibits *de novo* purine synthesis through competition with the purine derivatives hypoxanthine and guanine. Since 6-MP is converted into a toxic metabolite by hypoxanthine phosphoribosyltransferase encoded by *hpt*, inactivation of *hpt* results in 6-MP resistance in *E. coli* [3]. Three 6-MP evolved strains had mutations in *hpt* (Supplementary Data 4). Among the three strains, two strains (6-MPE4 and 6-MPE6) had additional mutations in *purR* encoding purine repressor PurR. We reconstructed *hpt* mutant strain carrying G40D mutation which is found in 6-MPE6, 6-MPE3, and 6-MPE4 strains. Since the reconstructed mutant strains showed amino acid auxotrophy, we added all 20 amino acids to the modified M9 medium to quantify 6-MP

resistance of the strain. As a result, we found that the *hpt* mutant showed an 18-fold increase in 6-MP resistance in comparison with the parent strain under the same condition. Although a reconstructed *purR* inactivation mutant did not show significantly increased 6-MP resistance, an epistatic interaction between *hpt* and *purR* mutations may increase 6-MP resistance. Although the mechanism by which 6-MP selects for purR deficient mutants is unknown, a previous study also reported the high frequency of inactivation of both *hpt* and *purR* double mutants among 6-MP resistant *E. coli* cells [3].

ABU evolved strains

DL-2-aminobutyric acid (ABU) is an α-amino acid that is an alanine derivative. The biological target in bacteria is unknown. Two ABU evolved strains had the same point mutation (P128Q) in *yfdZ* encoding glutamate-pyruvate aminotransferase (Supplementary Data 4). However, a reconstructed *yfdZ* mutant strain carrying the P128Q mutation did not show resistance to ABU. Therefore, the contributions of mutations in *yfdZ* to ABU resistance is unclear.

AF evolved strains

Acriflavine (AF) is an acridine derivative which interacts with DNA through intercalation. All four AF evolved strains had mutations in *acrR* encoding a repressor for *acrAB* and *ybjK* encoding DNA-binding transcriptional regulator RcdA (Supplementary Data 4). Reconstructed *acrR* and *ybjK* inactivation mutant strains showed 2.6-fold and 2.5-fold increased AF resistance, respectively (Supplementary Data 2). It was also reported that AcrAB is involved in AF resistance [4]. These results indicate that AF is pumped out by the AcrAB/TolC multi-drug efflux pump. Since YbjK is involved in the regulation of a number of stress response genes [5], it is possible that inactivation of *ybjK* changes the transcriptome to confer AF resistance. The reconstructed *ybjK* mutant strain also showed cross-resistance to CP and TET and collateral sensitivity to B-Cl-Ala (Supplementary Data 2).

ATP evolved strains

Amitriptyline hydrochloride (ATP) is a tricyclic antidepressant whose biological target in *E. coli* is unknown. Three ATP evolved strains had mutations in *acrR* encoding a repressor for *acrAB* (Supplementary Data 4). A reconstructed *acrR* inactivation mutant strain also showed a 2-fold increase in ATP resistance (Supplementary Data 2). These results strongly suggest that ATP is pumped out by the AcrAB/TolC multi-drug efflux pump.

AZT evolved strains

AZT is a monobactam antibiotic that specifically inhibits PBP3 encoded by *ftsI* in *E. coli* [6]. Common mutations identified in AZT evolved strains were *prlF* encoding the antitoxin for PrlF (SohA)-YhaV TA system (AZTE5 and AZTE4), *rssB* encoding a regulator of RpoS (AZTE5 and AZTE6), and *baeRS* encoding BaeRS two-component regulatory system (*baeS* in AZTE3 and *baeR* in AZTE5) (Supplementary Data 4). A reconstructed

prlF mutant strain carrying the duplication of TTCAACA sequences at 272 bp downstream of the start codon, *rssB* inactivation mutant strain, and *baeS* inactivation mutant strain showed 1.5-fold, 2.5-fold, and 1.4-fold increase in AZT resistance, respectively (Supplementary Data 2). Contributions of *prlF* and *rssB* mutations to AZT and other cell wall inhibitor resistances are described in the main text. Previous studies showed that overexpression of *baeRS* induces MdtABC multidrug exporter system and results in multidrug resistance [7,8]. Both AZTE3 and AZTE5 strain carrying either *baeS* or *baeR* mutation showed significantly increased *mdtABC* expression, which suggests the efflux of AZT by MdtABC (Supplementary Data 3).

B-Cl-Ala evolved strains

An alanine derivative B-Cl-Ala inhibits alanine racemase and alanine aminotransferase. Common mutations identified in B-Cl-Ala were *sstT* encoding sodium:serine/threonine symporter (B-Cl-AlaE1 and B-Cl-AlaE2), *rpoC* encoding β′ subunit of RNA polymerase (B-Cl-AlaE1 and B-Cl-AlaE2), and *livM* encoding leucine/isoleucine/valine transporter subunit (B-Cl-AlaE1 and B-Cl-AlaE6) (Supplementary Data 4). Reconstructed *rpoC* mutant strain carrying an 8 bp deletion of the 4172 to 4179 bp region downstream of the start codon (identified in B-Cl-AlaE1) and *livM* Q334R mutant strain (identified in both B-Cl-AlaE1 and B-Cl-AlaE6) showed 2.9-fold and 3.3-fold increases in B-Cl-Ala resistance, respectively, while a reconstructed *sstT* inactivation mutant strain did not show increased B-Cl-Ala resistance (Supplementary Data 2). These results strongly suggest the uptake of B-Cl-Ala by the LivMJHFKG transporter. These results also suggest that the *rpoC* mutations affect transcriptome resulting in resistance to B-Cl-Ala.

BSD evolved strains

Blasticidin S hydrochloride (BSD) is a nucleoside antibiotic that inhibits protein translation. All four BSD evolved strains had mutations in at least one of the *oppACF* genes encoding oligopeptide transporter subunits (*oppA* mutations in BSDE2 and BSDE6, *oppC* mutations in BSDE2 and BSDE5, and an *oppF* mutation in BSDE4) (Supplementary Data 4). A reconstructed *oppA* inactivation mutant strain showed 37-fold increased BSD resistance (Supplementary Data 2), which strongly suggested the uptake of BSD by the OppACF oligopeptide transporter. In addition, all four BSD evolved strains also had mutations in either *potA* or *potB* encoding polyamine transporter subunits (*potA* mutations in BSDE2, BSDE5, and BSDE6, a *potB* mutation in BSDE4) (Supplementary Data 4). A reconstructed *potA* inactivation mutant strain showed a 1.6-fold increased BSD resistance (Supplementary Data 2). PotABCD is a spermidine preferential ABC transporter. Spermidine and other polyamines are known to bind RNA and thereby affect translation in *E. coli* [9]. Since *potACD* mutations were also identified in all four PLM evolved strains and the reconstructed *potA* mutant strain also showed 2.9-fold increase in PLM resistance (Supplementary Data 2), these results indicate that the inactivation of the polyamine transporter confers multidrug resistance including BSD and PLM.

BZ evolved strains

Benserazide hydrochloride (BZ) is an aromatic L-amino acid decarboxylase inhibitor which is used in the management of Parkinson's disease. The BZ mechanism of action in bacteria is unclear. Among the six independent culture lines, only two lines showed significant increases in BZ resistance and the two BZ evolved strains (BZE1 and BZE2) had the same frameshift mutation in *pstB* encoding a phosphate transporter subunit (Supplementary Data 4). In the supervised PCA space, these two evolved strains were classified as class 3 and these two strains showed a significant increase in *yibD* and *yibA* expression (Fig. 2d). Our attempts to reconstruct a *pstB* mutant strain carrying the same frameshift mutation failed. Contributions of *pstB* mutations to BZ resistance is unclear. Interestingly, these two BZ evolved strains also had mutations in elongasome component genes, i.e. *mreB* and *rodZ*, and showed round in cell shape (data not shown).

CBPC evolved strains

CBPC is a β-lactam antibiotic which interacts with all PBPs [10]. Three CBPC evolved strains had the same *prlF* mutation (duplication of TTCAACA sequences at 272 bp downstream of the start codon) and the reconstructed *prlF* mutant strain showed a 2.0-fold increase in CBPC resistance (Supplementary Data 2). Contributions of the *prlF* mutation to CBPC and other cell wall inhibitor resistances are described in the main text.

CCCP evolved strains

Carbonyl cyanide 3-chlorophenylhydrazone (CCCP) is an uncoupling agent that inhibits oxidative phosphorylation. All four CCCP evolved strains had mutations in *mprA* encoding a repressor for the multidrug resistance pump EmrAB (Supplementary Data 4). These evolved strains, except for the CCCPE3 strain, were categorized in class 1 (Fig. 2d). The CCPE3 strain had an additional mutation in the *barA* encoding two-component regulatory system with UvrY (Supplementary Data 4), therefore this strain showed a different transcriptome from the other CCCP evolved strains. Consistent with this study (Fig. 2B), it was also reported that a mutation in the *mprA* gene results in EmrAB overexpression and increased resistance to CCCP [11]. A reconstructed *mprA* inactivation mutant strain also showed 1.6-fold increased resistance to CCCP (Supplementary Data 2). These results indicate the efflux of CCCP by the EmrAB pump.

CMZ evolved strains

CMZ is a second-generation cephalosporin that inhibits all PBPs except PBP2 in *E. coli* [12]. No common mutation was identified in CMZ evolved strains. One CMZ evolved strain (CMZE4) had mutations in *ompR* and *envZ* encoding OmpR/EnvZ two-component system and another CMZ evolved strain (CMZE6) had a mutation in *prlF*. The *prlF* mutation is the same as in other evolved strains carrying a *prlF* mutation, i.e. a duplication of TTCAACA sequences at 272 bp downstream of the start codon (Supplementary Data 4). CMZ is a second-generation cephalosporin and both *prlF* and *ompF* mutations were also found in evolved strains to monobactam AZT and penicillin antibiotics CBPC and MEC (Supplementary Data 4). The reconstructed *ompF* inactivation mutant strain showed increased resistance to MEC (Supplementary Data 2), strongly suggesting uptake

of CMZ by the OmpF porin. On the other hand, the reconstructed *prlF* mutant strain carrying a duplication of the TTCAACA mutation did not show an increase in CMZ resistance.

CP evolved strains

CP is an amphenicol antibiotic that specifically binds to 23S rRNA of the 50S ribosomal subunit. Three CP evolved strains (CPE2, CPE3, and CPE6) had mutations in *acrR*. Among the three strains, CPE2 and CPE6 strains additionally had mutations in *rssB* (Supplementary Data 4). The reconstructed *acrR* and *rssB* inactivation mutant strains showed 1.6-fold and 1.9-fold increases in CP resistance, respectively (Supplementary Data 2). CPE6 and CPE5 strains also had mutations in either *ompR* or *ompF*, respectively. The reconstructed *ompF* inactivation mutant strain showed a 2.1-fold increase in CP resistance (Supplementary Data 2).

DCS evolved strains

D-cycloserine (DCS) is an amino-acid derivative which inhibits D-alanyl-D-alanine ligases A and B, and alanine racemase [13]. Three DCS evolved strains (DCSE3, DCSE4, and DCSE5) had mutations in *cycA* encoding D-alanine/D-serine/glycine transporter (Supplementary Data 4). DCSE2 and DCSE5 strains had mutations in *dadA* encoding D-amino acid dehydrogenase (Supplementary Data 4). Previous studies showed that CycA transports DCS into *E. coli* cells [13,14]. It also suggested that DadA modifies DCS into a more potent antibiotic; therefore, a *dadA* mutation confers DCS resistance [14]. A reconstructed *cycA* inactivation mutant strain and a reconstructed *dadA* mutant strain carrying G9S mutation, which was found in DCSE5 strain, showed 37-fold and 12-fold increases in DCS resistance, respectively (Supplementary Data 2). DCSE2 and DCSE5 strains also had the same *prlF* mutation (duplication of TTCAACA sequences at 272 bp downstream of the start codon) and the reconstructed *prlF* mutant strain showed a 1.6-fold increase in DCS resistance (Supplementary Data 2). Contributions of the *prlF* mutation to DCS and other cell wall inhibitor resistances are described in the main text.

DVAL evolved strains

Although the mechanism of action of DVAL is unknown in *E. coli*, it was reported that DVAL affects extracellular polysaccharide production resulting in the inhibition of biofilm formation in *Porphyromonas gingivalis* [15]. Two DVAL evolved strains (DVALE6 and DVALE5) had mutations in *yhjE* encoding predicted transporter (Supplementary Data 4). DVALE1 and DVALE5 strains had mutations in the same *prlF* mutation (duplication of TTCAACA sequences at 272 bp downstream of the start codon) (Supplementary Data 4). A reconstructed *yhjE* inactivation mutant strain and the reconstructed *prlF* mutant strain showed 2.7-fold and 2.3-fold increased DVAL resistance, respectively (Supplementary Data 2). Contributions of the *yhjE* and the *prlF* mutations to DVAL resistance are described in the main text. DAVLE6 and DVALE2 strains also had mutations in *gshA* encoding gamma-glutamate-cysteine ligase (Supplementary Data 4). A reconstructed *gshA* inactivation mutant strain showed a 6.0-fold increase in DVAL

resistance (Supplementary Data 2). Positive effects of *gshA* overexpression on thiol-specific damage and some metal tolerance were reported [16,17], while the effect of *gshA* inactivation on stress resistance was unclear. The reconstructed *gshA* inactivation mutant strain showed cross-resistance to several chemicals e.g. CP, PLM, NVA, TET, sodium salicylate (SS), BSD, DVAL, and 5-FOA and collateral sensitivity to B-Cl-Ala and NQO (Supplementary Data 2). Mutations in *gshA* were also identified in other evolved strains i.e. two L-glutamic acid γ-hydrazide (GAH) evolved strains (GAHE1 and GAHE3) and one DCS evolved strain (DCSE2) (Supplementary Data 4). Interestingly, our transcriptome analysis revealed that these evolved strains carrying the *gshA* mutation commonly showed a 2.1- to 16.6-fold increase in *gcvB* expression (Supplementary Data 3). GcvB is a small RNA which is involved in the regulation of amino acid availability [18]. These results suggest that GcvB overexpression mediated by *gshA* mutation results in alteration of chemical resistance, including DVAL resistance.

EDTA evolved strains

EDTA is a metal chelator which also disrupts the cell wall structure in Gram-negative bacteria [19]. Two EDTA evolved strains had a mutation in *secB* encoding protein export chaperone (Supplementary Data 4). One evolved strain (EDTAE1) had a point mutation (I13S) and the other strain (EDTAE3) had a nonsense mutation (Y56*, TAC→TAA). Our attempts to reconstruct the *secB* mutant strain carrying either I13S or Y56* mutation failed. Contributions of mutations in *secB* to EDTA resistance remain unclear.

EM evolved strains

EM is a macrolide antibiotic which binds to the bacterial P site on the 50S ribosomal subunit. Three EM evolved strains (EME1, EME3, and EME6) have mutations in *ycbZ* encoding predicted peptidase (Supplementary Data 4). A reconstructed *ycbZ* inactivation mutant strain showed a 2.6-fold increase in EM resistance (Supplementary Data 2). The contribution of the *ycbZ* mutation to EM resistances is described in the main text. EME2 and EME6 strains also had the same point mutations in the intergenic region between *rplM* encoding 50S ribosomal subunit protein L13 and *yhcM* encoding a conserved protein with nucleoside triphosphate hydrolase domain (at 155 bp upstream of the *rplM* start codon) (Supplementary Data 4). A reconstructed mutant strain carrying the same point mutation showed a 1.4-fold increased EM resistance (Supplementary Data 2).

FOS evolved strains

Fosfomycin disodium salt (FOS) is a phosphonic antibiotic that inhibits UDP-*N*-acetylglucosamine-3-enolpyruvyltransferase encoded by *murA* gene. Two FOS evolved strains (FOSE3 and FOSE6) had mutations in *glpT* encoding sn-glycerol-3-phosphate transporter, while the two other FOS evolved strains (FOSE1 and FOSE5) had mutations in *cyaA* encoding adenylate cyclase (Supplementary Data 4). Reconstructed *glpT* and *cyaA* inactivation mutant strains showed 12.9-fold and 5.4-fold increase in FOS resistance, respectively (Supplementary Data 2). GlpT is known to import FOS, and the *cyaA* mutation is also known to regulate GlpT activation resulting in FOS resistance in *E. coli*

[20,21].

FTD evolved strains

Furaltadone (FTD) is an oxazolidinone that targets DNA, ribosomes, and many macromolecules. All four FTD evolved strains had mutations in *nfsA* encoding NADPH-dependent nitroreductase A (Supplementary Data 4). Mutations in *acrR* and *ompR*/*envZ* were identified in three FTDE evolved strains (*acrR* in FTDE1, FTDE3, and FTDE4, *ompR* in FTDE1 and FTDE3, *envZ* in FTDE2) (Supplementary Data 4). FTDE2 and FTDE3 strains also had mutations in *ompF* (Supplementary Data 4). Reconstructed *nfsA*, *acrR*, and *ompF* inactivation mutant strains showed 3.8-fold, 1.6-fold, and 3.6-fold increases in FTD resistance, respectively (Supplementary Data 2). To show an antibiotic effect, nitrofurans including FTD and nitrofurantoin (NIT) need to be nitroreducted by NfsA or NfsB [22,23]. All four NIT evolved strains in this study also had mutations in *nfsA* and the reconstructed *nfsA* mutant strain also showed a 4.3-fold increase in NIT resistance (Supplementary Data 2). Contributions of *acrR* and *ompF* mutations to FTD resistance are described in the main text.

GAH evolved strains

GAH is a glutamate analog which is known to inhibit glutamate decarboxylase and γ-aminobutyrate aminotransferase. As described in the main text, the highest number of mutations were observed in GAH evolved strains carrying between 73 to 223 mutations, implying that GAH is a mutagen. Thirty-eight genes were identified as common mutations in GAH evolved strains (Supplementary Data 4). Among the genes, we selected six genes (*glnP* encoding a glutamine transporter subunit, *metN* encoding a DL-methionine transporter subunit, *mprA*, *sdaA* encoding l-serine deaminase I, *gshA*, and *gadB* encoding glutamate decarboxylase B) and constructed *glnP*, *metN*, *mprA*, *sdaA*, and *gshA* inactivation mutant strains and a *gadB* mutant strain carrying D39N mutation. The reconstructed *glnP*, *metN*, *mprA* inactivation mutant strains showed 8.4-fold, 1.8-fold, and 12.9-fold increased GAH resistance respectively (Supplementary Data 2). These results strongly suggest that GAH is mainly imported by the GlnPQ transporter and the EmrAB/TolC multidrug efflux pump which is repressed by MprA. On the other hand, the reconstructed *sdaA*, *gshA*, and *gadB* mutant strains did not show increased GAH resistance (Supplementary Data 2).

H_2O_2 evolved strains

H_2O_2 is the simplest peroxide which acts as a reactive oxygen species and damages DNA, proteins, and membrane lipids. Among the six independent culture lines, only two lines showed a significant increase in H_2O_2 resistance; these two H_2O_2 evolved strains had mutations in *oxyR* encoding a positive regulator of H_2O_2-inducible genes in *E. coli* (Supplementary Data 4) [24]. A reconstructed *oxyR* mutant strain (A147E mutation) also showed a 4-fold increased resistance to H_2O_2 (Supplementary Data 2). The reconstructed *oxyR* mutant strain showed cross-resistance to BZ and collateral sensitivity to nickel (II) chloride ($NiCl_2$) and potassium tellurite (IV) (K_2TeO_3).

HSE evolved strains

L-homoserine (HSE) is the precursor of both threonine and methionine. In *E. coli*, HSE is known to inhibit $NADP^+$-specific glutamate dehydrogenase, which is the first enzyme in ammonia assimilation [25]. Three HSE evolved strains had mutations in *cycA* encoding D-alanine/D-serine/glycine transporter and a reconstructed *cycA* inactivation mutant strain also showed a 1.5-fold increase in HSE resistance (Supplementary Data 2). These results strongly suggest the uptake of HSE by CycA.

KM evolved strains

Kanamycin sulfate (KM) is an aminoglycoside antibiotic which targets bacterial 30S ribosome. Three KM evolved strains (KME1, KME4, and KME6) had mutations in the *fusA* encoding protein chain elongation factor EF-G (Supplementary Data 4). In addition, all four KM evolved strains had mutations in genes related to the electron transport chain i.e. *cyoE* in KME1, *nuoG* in KME4, *cyoA* in KME5, and *cyoB* in KME6 (Supplementary Data 4). KME5 and KME6 strains also had mutations in *oppA* encoding the oligopeptide transporter subunit (Supplementary Data 4). A reconstructed *fusA* mutant strain carrying the P659L mutation, which was found in KME1, showed a 6.0-fold increase in KM resistance (Supplementary Data 2). Reconstructed *cyoA* and *cyoB* inactivation mutant strains also showed 2.2-fold and 1.9-fold increases in KM resistance, respectively (Supplementary Data 2). Previous studies also reported that mutations in *fusA* and genes related to the electron transport chain confer aminoglycoside resistance [26–28]. It was shown that proton motive force is required for bacterial aminoglycoside uptake [29]; therefore, mutations in the genes related to the electron transport chain confer aminoglycoside resistance [26]. Mogre et al. also showed that inactivation of *cyaA* encoding adenylate cyclase results in KM resistance [28]; we also confirmed that a reconstructed *cyaA* inactivation mutant strain showed a slightly increased KM resistance of 1.3-fold (Supplementary Data 2). A reconstructed *oppA* inactivation mutant strain did not show significantly increased KM resistance (Supplementary Data 2). Interestingly, a previous study also reported that mutations in *oppA* were found in *E. coli* aminoglycoside resistance strains [30], while deletion of the *oppA* gene did not confer aminoglycoside resistance [31]. Thus, the contribution of *oppA* mutation in KM resistance is unclear.

K_2TeO_3 evolved strains

K_2TeO_3 is highly toxic for most microorganism, including *E. coli*, due to its activity as a strong oxidizing agent over many macromolecules [32]. No common mutation was identified in K_2TeO_3 evolved strains. One K_2TeO_3 evolved strain (KTeE5) had a mutation in *rne* encoding RNase E. Although *rne* is an essential gene in *E. coli*, its C-terminus, which is a protein scaffold region, is non-essential [33]. The KTeE5 strain had a frameshift mutation which resulted in disruption of the C-terminal domain. A reconstructed *rne* mutant strain carrying the same frameshift mutation showed a 3-fold increase in K_2TeO_3 resistance. Since RNase E plays an important role in global mRNA metabolism [34], these results suggest that the expression of genes involved in K_2TeO_3 resistance is regulated by

RNase E. Interestingly, the reconstructed *rne* mutant strain showed cross-resistance to CP, AZT, CBPC, K_2TeO_3, and EM, and collateral sensitivity to H_2O_2, 6-MP, 3-AT, and GAH (Supplementary Data 2).

LVAL evolved strains

The *E. coli* K-12 strain has two functional acetohydroxy acid synthases (AHAS) encoded by *ilvBN* and *ilvIH*, which catalyze the initial step of LVAL and isoleucine (Ile) biosynthesis [35]. The activities of these enzymes are subject to feedback inhibition by LVAL [36], and increased intracellular concentrations of LVAL cause Ile depletion [37]. All four strains in class 7 are LVAL evolved strains and these strains had mutations in RNA degradosome components i.e. *rhlB* encoding an RNA helicase (3 out of 4) or *rne* encoding an RNase E (the other strain) (Fig. 2e and Supplementary Data 4). RNase E is an essential RNase and plays an important role in mRNA metabolism together with RhlB, which interacts with the non-essential C-terminus of RNase E [33]. Our transcriptome analysis revealed that all of the LVAL evolved strains showed increased expressions of *ilvBN*. This result is reasonable since LVAL inhibits *ilvBN* products resulting in Ile depletion. In all class 7 strains, *rhlB* or *rne* were commonly mutated; this suggests regulation of *ilvBN* mRNA levels by the RNA degradosome. However, both reconstructed *rhlB* mutant strains carrying either an inactivation mutation or G120C mutation, which is found in LVAL evolved strain LVALE3, and the *rne* mutant strain carrying C-terminus disruption mutation did not show LVAL resistance. Therefore, the contributions of mutations in the RNA degradosome components to LVAL resistance is unclear. These LVAL evolved strains commonly showed cross resistances to β-chloro-l-alanine (B-Cl-Ala), potassium tellurite (K_2TeO_3), and fosfomycin (FOS), and collateral sensitivities to 6-MP and GAH (Fig. 2e).

MEC evolved strains

Mecillinam (MEC) is a penicillin antibiotic that specifically inhibits PBP2 encoded by *mrdA* in *E. coli* [38]. No common mutation was identified in MEC evolved strains. One MEC evolved strain (MECE5) had a mutation in *prlF* and another MEC evolved strain (MECE6) had a mutation in *ompF*. The *prlF* mutation is the same as other evolved strains carrying *prlF* mutation i.e. duplication of TTCAACA sequences at 272 bp downstream of the start codon (Supplementary Data 4). Both *prlF* and *ompF* mutations were also found in evolved strains to another penicillin antibiotic CBPC and monobactam AZT (Supplementary Data 4). The reconstructed *prlF* mutant strain carrying a duplication of the TTCAACA mutation and the *ompF* inactivation mutant strain showed increased resistance to MEC, which strongly suggests uptake of MEC by the OmpF porin.

MMC evolved strains

MMC is an alkylating agent that induces a SOS response. Since MMC is a DNA crosslinker, the MMC evolved strains carried a relatively high number of mutations (between 22 to 31). All four MMC evolved strains had mutations in *acrR* and the reconstructed *acrR* inactivation mutant strain showed a 1.9-fold increase in MMC

resistance (Supplementary Data 2). These results strongly suggest that MMC is pumped out by the AcrAB/TolC multi-drug efflux pump. Interestingly, all four MMC evolved strains also had mutations in *sulA* encoding SOS cell division inhibitor (Supplementary Data 4). A reconstructed *sulA* inactivation mutant strain showed 2.8-fold increased MMC resistance (Supplementary Data 2). These results indicate that inhibition of the SOS response results in MMC resistance. Two MMC evolved strain (MMCE1 and MMCE4) had mutations in the intergenic region between *ogt* encoding methylated-DNA-[protein]-cysteine S-methyltransferase and *hrpA* encoding a DEAH-box RNA helicase intergenic region (at 172 bp upstream of the *ogt* start codon) (Supplementary Data 4). A reconstructed mutant strain carrying the point mutation in *ogt*/*hrpA* intergenic region, which was found in MMCE4, showed a 3.8-fold increase in MMC resistance (Supplementary Data 2). A previous study showed that the deletion of the *ogt* gene results in increased sensitivity to alkylating agents and overexpression of *ogt* results in increased resistance to alkylating agents [39]. Two genes, *yjcO* encoding a conserved protein of unknown function and s*oxR* encoding a redox-sensing transcriptional dual regulator, were also identified as common mutations among the MMC evolved strains (mutations in *yjcO* in MMCE4 and MMCE6, and mutations in *soxR* in MMCE4 and MMCE5 were identified) (Supplementary Data 4). However, reconstructed mutant strains carrying either the *yjcO* L84L (CTG→CTT) mutation, which was found in MMCE4, or *soxR* inactivation mutation did not show significant MMC resistance (Supplementary Data 2). Contributions of the *yjcO* and *soxR* mutations to MMC resistance is unclear.

NFLX evolved strains

NFLX is a fluoroquinolone that inhibits the bacterial DNA gyrase. All four NFLX evolved strains had mutations in *ompF* and the reconstructed *ompF* inactivation mutant strain showed a 5.1-fold increase in NFLX resistance (Supplementary Data 2). Three NFLX evolved strains also had mutations in *gyrA* encoding DNA gyrase (type II topoisomerase) subunit A and a reconstructed *gyrA* mutant strain carrying the H45Y mutation, which was identified in the NFLXE1 strain, showed a 1.7-fold increase in NFLX resistance (Supplementary Data 2). The contribution of *ompF* and *gyrA* mutations to NFLX resistance is described in the main text. Two NFLX evolved strains (NFLXE1 and NFLXE4) also had mutations in *mipA* encoding a scaffolding protein for murein synthesizing machinery (Supplementary Data 4). A previous study reported that MipA is involved in antibiotic resistance and deletion of *mipA* resulted in a slight increase in nalidixic acid (an original quinolone antibiotic) and streptomycin resistances [40]. However, a reconstructed *mipA* inactivation mutant strain did not show increased NFLX resistance in our study.

$NiCl_2$ evolved strains

$NiCl_2$ causes oxidative stress that can damage proteins, DNA, and lipids. Three $NiCl_2$ evolved strains had the same nonsense mutation (E38*, GAG→TAG) in *corA* encoding the magnesium/nickel/cobalt transporter (Supplementary Data 4). A reconstructed *corA* inactivation mutant strain also showed an 8-fold increase in $NiCl_2$ resistance, indicating

$NiCl_2$ resistance by blocking Ni^{2+} uptake.

NIT evolved strains

NIT is a nitrofuran derivative hydantoin that targets DNA, ribosomes, and other many macromolecules. All four NIT evolved strains had mutations in *nfsA* and the reconstructed *nfsA* inactivation strain showed a 4.3-fold increase in NIT resistance (Supplementary Data 2). The contribution of *nfsA* mutation to NIT resistance is described in the results of FTD resistance. Two NIT evolved strains (NITE1 and NITE3) also had mutations in *mprA* and the *mprA* inactivation mutant strain showed a 1.5-fold increase in NIT resistance (Supplementary Data 2). The contribution of *mprA* mutation to NIT resistance is described in the main text.

NMNO evolved strains

NMNO is an amine compound whose biological target in *E. coli* is unknown. Two NMNO evolved strains (NMNOE5 and NMNOE6) had mutations in *ycbZ* and the reconstructed *ycbZ* inactivation mutant strain showed a 2.1-fold increase in NMNO resistance (Supplementary Data 2). Contributions of the *ycbZ* mutation to NMNO resistance is described in the main text. Two genes, *msbA* encoding a lipid A-core flippase and *lon* encoding Lon protease, were also identified as common mutations among the NMNO evolved strains (mutations in *msbA* in NMNOE1 and NMNOE5, and mutations in *lon* in NMNOE4 and NMNOE6 were identified) (Table SX4). A reconstructed *lon* mutant strain carrying the A236D mutation, which was found in the NMNOE6 strain, showed slightly increased (1.3-fold) NMNO resistance, while a reconstructed *msbA* mutant strain carrying the L171W mutation which was found in NMNOE1 did not show NMNO resistance (Supplementary Data 2). A previous study showed that the *lon* mutation induces AcrAB/TolC multi-drug efflux pump via stabilization of the *acrAB* transcriptional activators MarA and SoxS [41]. We also confirmed that the reconstructed *acrR* inactivation mutant strain showed 1.5-fold increased NMNO resistance (Supplementary Data 2). These results strongly suggest that NMNO is pumped out by the AcrAB/TolC multi-drug efflux pump. Contributions of the *msbA* mutation to NMNO resistance is unclear.

NQO evolved strains

NQO is a mutagen that induces G:C to A:T transitions, G:C to T:A transversions, and frameshifts [42]. The NQO evolved strains carried a relatively high number of mutations (between 17 to 30). All four NQO-evolved strains had mutations in both *acrB* and *acrR* genes, and the reconstructed *acrR* inactivation mutant strain showed a 1.3-fold increase in NQO resistance (Supplementary Data 2). These results suggest that NQO is pumped out by the AcrAB/TolC multi-drug efflux pump. NOQE4 and NQOE5 strains also had mutations in the *ydhO* encoding predicted lipoprotein/*sodB* encoding superoxide dismutase (Fe) intergenic regions (at 3 bp or 95 bp upstream of the *sodB* start codon respectively) (Supplementary Data 4). Our transcriptome analysis also confirmed that NQOE4 and NQOE5 strains showed 2.1- and 3.4-fold increased *sodB* expression

respectively (Supplementary Data 3). A reconstructed mutant strain carrying G→T mutations at 95 bp upstream of the *sodB* start codon showed a 2.0-fold increase in NQO resistance (Supplementary Data 2). It was reported that *E. coli* lacking superoxide dismutase showed significant sensitivity to DNA oxidation [43], and overexpression of *sodB* reduced mutation frequency [44]. These results indicate that the *sodB* overexpression results in suppression of DNA damage and an increase in NQO resistance.

NVA evolved strains

NVA is a threonine analog that inhibits aspartate kinase, homoserine dehydrogenase, and homoserine kinase. Two NVA evolved strains (NVAE1 and NVAE2) had mutations in *sstT* encoding sodium: serine/threonine symporter and the reconstructed *sstT* inactivation mutant strain showed a 6.9-fold increase in NVA resistance (Supplementary Data 2). These results strongly suggest the uptake of NVA by SstT. The NVAE1 and NVAE2 also had mutations in *glyT* encoding tRNA-Gly(UCC) and a reconstructed *glyT* mutant strain carrying G→A mutation at 30 bp downstream of the mature 5′-end and showed a 3.0-fold increase in NVA resistance (Supplementary Data 2). NVAE5 and NVAE6 strains had mutations in either *thrA* encoding fused aspartokinase I and homoserine dehydrogenase I (G403D mutation) or *dapA* encoding dihydrodipicolinate synthase (P105S mutation) (Supplementary Data 4). Since ThrA is subjected to feedback inhibition by threonine [45], the point mutation in *thrA* probably outwits the feedback inhibition resulting in NVA resistance. DapA converts L-aspartate 4-semialdehyde (ASA) to (2S,4S)-4-hydroxy-2,3,4,5-tetrahydrodipicolinate. Since ASA is also converted to L-homoserine by ThrA, the point mutation in *dapA* may increase the L-homoserine pool, resulting in NVA resistance.

PHEN evolved strains

1,10-phenanthroline monohydrate (PHEN) is a *N*, *N*′-heterocyclic chelating ligand showing antimicrobial properties. All PHEN evolved strains had mutations in *acrR* (Supplementary Data 4). The reconstructed *acrR* inactivation mutant strain also showed a 1.5-fold increase in PHEN resistance. These results indicate that PHEN is pumped out by the AcrAB/TolC multi-drug efflux pump.

PLM evolved strains

PLM, also known as zeocin, is a bleomycin family antibiotic that intercalates DNA resulting in DNA double-strand break. The PLM evolved strains carried a relatively high number of mutations (between 14 to 18). All four PLM evolved strains had mutations in *cyoB* and the reconstructed *cyoB* inactivation mutant strain showed a 3.8-fold increase in PLM resistance (Supplementary Data 2). We also confirmed that mutations in other genes related to the electron transport chain conferred PLM resistance. The *cyoE* and *cyoA* inactivation mutant strains showed 3.4-fold and 3.5-fold increases in PLM resistance, respectively (Supplementary Data 2). Similar to aminoglycoside uptake, these results strongly suggest that proton motive force is required for bacterial PLM uptake. All four PLM evolved strains also had mutations in *potACD* genes encoding polyamine

transporter subunits (*potA* mutations in PLME1, PLME3, and PLME6, a *potC* mutation in PLME5, and a *potD* mutation in PLME1) (Supplementary Data 4). The reconstructed *potA* inactivation mutant strain showed a 2.9-fold increase in PLM resistance (Supplementary Data 2). Contribution of the polyamine transporter to PLM resistance is described in the part of BSD evolved strains in this supplementary text. Three PLM evolved strains had mutations in the *ptsP* encoding mannose-specific PTS enzyme IIC component and a reconstructed *ptsP* inactivation mutant strain showed a 1.9-fold increase in PLM resistance (Supplementary Data 2). Interestingly, the reconstructed *ptsP* inactivation mutant strain also showed a 2.6-fold increase in NVA resistance (Supplementary Data 2). These results suggest that inactivation of *ptsP* confers multidrug resistance. The other common mutations in PLM evolved strains were *rpoD* encoding sigma 70 (sigma D) factor (found in PLM1 and PLME3), *rfe* encoding UDP-*N*-acetylglucosamine undecaprenyl-phosphate *N*-acetylglucosaminephosphotransferase (found in PLME5 and PLME6), and *iscR* encoding an iron-sulfur cluster regulator (found in PLME1 and PLME6) (Supplementary Data 4). A reconstructed *rfe* inactivation mutant strain showed a 1.8-fold increase in PLM resistance (Supplementary Data 2). A reconstructed *iscR* mutant strain carrying the A155T mutation, which was found in the PLME6 strain, showed a slightly increased PLM resistance of 1.4-fold (Supplementary Data 2). The major Fe-S cluster biosynthesis machinery is encoded by the *iscRSUA* operon and the Fe-S cluster biosynthesis controls the uptake of aminoglycosides [29]. Since IscR represses expression of the *iscRSUA* operon [46], it suggests that the *iscR* point mutation negatively affects the Fe-S cluster biosynthesis machinery.

PMZ evolved strains

Promethazine hydrochloride (PMZ) is a phenothiazine which is a histamine H1 receptor inhibitor. It is shown that a phenothiazine derivative thioridazine is highly effective against *Mycobacterium tuberculosis* due to its multiple mechanisms of action, including efflux pump inhibiting activity, alteration of cell-envelope permeability, and inhibition of type-II NADH-menaquinone oxidoreductase (NDH-2) [47]. All four PMZ evolved strains had mutations in *acrR* and three PMZ evolved strains additionally had *acrA* mutations (Supplementary Data 4). The reconstructed *acrR* inactivation mutant strain also showed a 2-fold increase in PMZ resistance. These results indicate that PMZ is pumped out by the AcrAB/TolC multi-drug efflux pump.

PS evolved strains

PS is a highly cationic polypeptide that was shown to target the cytoplasmic membrane in *Salmonella typhimurium* [48]. No common mutation was identified in PS evolved strains.

PUR evolved strains

Puromycin dihydrochloride (PUR) is an aminonucleoside antibiotic that inhibits protein translation. Two PUR evolved strains (PURE2 and PURE3) had mutations in *soxR* encoding a redox-sensing transcriptional dual regulator (Supplementary Data 4). The PURE3 strain and another PUR evolved strain (PURE1) also had mutations in *acrR*

(Supplementary Data 4). Reconstructed *soxR* and *acrR* inactivation mutant strains showed a 1.2-fold and 2.5-fold increase in PUR resistance, respectively (Supplementary Data 2). It was also reported that AcrAB/TolC multi-drug efflux pump confers resistance to PUR [49]. Although the effect of *soxR* inactivation on PUR resistance was not so significant in this study, a previous study showed that mutations in *soxR* resulted in increased expression of AcrAB/TolC multi-drug efflux pump [50]. These results indicate that PUR is pumped out by the AcrAB/TolC multi-drug efflux pump.

RFP evolved strains

RFP is an antibiotic that inhibits DNA-dependent RNA polymerase in bacteria. No common mutation was identified in RFP evolved strains. One strain (RFPE4) had mutations in *acrB* gene (L931Q and P40T mutations) and the reconstructed *acrR* inactivation mutant strain showed a 1.5-fold increase in RFP resistance, indicating that RFP is pumped out by the AcrAB/TolC multi-drug efflux pump (Supplementary Data 2). The contribution of AcrAB/TolC to RFP resistance was also previously shown [51].

SDC evolved strains

Sodium dichromate dihydrate (SDC) induces chromate stress, which causes oxidative DNA damage and SOS response in *E. coli* [52]. All four SDC evolved strains had mutations in the *cysP* encoding thiosulfate transporter subunit (Supplementary Data 4). Except for the SDCE1 strain, the other SDC evolved strains had additional mutations in the *folM* encoding dihydrofolate reductase isozyme (Supplementary Data 4). In addition, SDCE2 and SDCE3 strains commonly had mutations in *frlA* encoding the predicted fructoselysine transporter and *rhlB* encoding a DEAD-box family RNA helicase (Supplementary Data 4). A reconstructed *folM* inactivation mutant strain showed a 1.5-fold increase in SDC resistance. However, reconstructed mutant strains carrying the V323M mutation, which was found in both SDCE2 and SDCE3 strains, *cysP* inactivation mutation, or *rhlB* inactivation mutation, did not show increased SDC resistance (Supplementary Data 2). Since the reconstructed *cysP* inactivation mutants strain showed amino acid auxotrophy, the IC_{50} values of the *cysP* mutant strain were determined in the modified M9 medium supplemented with all 20 amino acids. Although SDCE2 and SDCE3 strains had the same frameshift mutation in *cysP* (Supplementary Data 2), these evolved strains could grow on the modified M9 minimal medium. It is possible that combinations of *cysP* and the other mutations rescue the amino acid auxotrophy and results in increased SDC resistance.

SHX evolved strains

DL-serine hydroxamate (SHX) is a serine analog that inhibits serine-tRNA ligase and induces a stringent response. All four SHX evolved strains had mutations in *gabP* encoding gamma-aminobutyrate transporter and a reconstructed *gabP* inactivation mutant strain showed a 2.0-fold increase in SHX resistance (Supplementary Data 2). These results strongly suggest the uptake of SHX by GabP. Two SHX evolved strains (SHXE1 and SHXE4) additionally had mutations in *serA* encoding D-3-phosphoglycerate dehydrogenase. A reconstructed serA mutant strain carrying the V379M mutation, which

was found in the SHXE1 strain, showed a 1.5-fold increase in SHX resistance (Supplementary Data 2). It was reported that D-3-phosphoglycerate dehydrogenase, which is the first enzyme in the biosynthetic pathway to serine and glycine, is subject to feedback inhibition by serine [53]. Therefore, the mutations in *serA* result in SHX resistance due to the prevention of feedback inhibition.

SS evolved strains

It was reported that SS inhibits Enterobacteriaceae *Klebsiella pneumoniae* cell growth and the production of capsular polysaccharide [54]. SS is also known to inhibit motility and biofilm production of *E. coli* thorough decreases in the expression of flagella proteins [55,56]. Since SS has a chelating property, SS is thought to inhibit enzymes directly involved in polysaccharide synthesis and outer membrane stabilization through chelation of divalent cations used for these enzymes [54]. Three SS evolved strains (SSE1, SSE4, and SSE6) had the same point mutation in *sdhA* encoding succinate dehydrogenase and the flavoprotein subunit (C257F mutation) (Supplementary Data 4). Common mutations in the *ilv* operon were also identified in three SS evolved strains (*ilvL* encoding *ilvXGMEDA* operon leader peptide in SSE1, and *ilvE* encoding branched-chain amino-acid aminotransferase in SSE2 and SSE4) (Supplementary Data 4). A reconstructed *sdhA* mutant strain carrying C257F mutation showed a 2.3-fold increase in SS resistance, while a reconstructed *ilvL* inactivation mutant strain did not show increased SS resistance (Supplementary Data 2). It was reported that salicylic acid stimulates the ubiquinone binding site of mitochondrial SDH [57]. These results suggest that alteration of SDH activity reduces SS toxicity.

SXZ evolved strains

SXZ is a sulfonamide that inhibits folic acid biosynthesis. Three SXZ evolved strains (SXZE1, SXZE2, and SXZE4) had mutations in *folM* encoding dihydromonapterin reductase, while the other strain (SXZE5) had mutations in *folX* encoding dihydroneopterin triphosphate 2'-epimerase (Supplementary Data 4). A reconstructed *folM* inactivation mutant strain showed a 2.2-fold increase in SXZ resistance (Supplementary Data 2). A previous study also showed that disruption of either *folM* or *folX* results in increased resistance to the folic acid biosynthesis inhibitors trimethoprim and sulfamonomethoxine [58]. On the other hand, it was suggested that *folX* mutations result in metabolic compensation by increasing the flux of metabolites through folic acid biosynthesis [58]. Two SXZ evolved strains (SXZE2 and SXZE5) had mutations in *mprA* (Supplementary Data 4). Although the reconstructed *mprA* inactivation mutant strain did not show increased resistance to SXZ, the evolved strains in class 1, which commonly had mutations in *mprA*, tended to show increased resistance to SXZ (Supplementary Data 2, Fig 2e). The contribution of the EmrAB/TolC multi-drug efflux pump to SXZ resistance is unclear.

TET evolved strains

TET is a protein synthesis inhibitor that binds to the bacterial 30S ribosome. All four TET evolved strains had mutations in *acrR* and the reconstructed *acrR* inactivation mutant

strain showed a 4.1-fold increase in TET resistance (Supplementary Data 2). TETE2 and TETE4 had mutations in either *envZ* or *ompR*, respectively, and the reconstructed *ompF* inactivation mutant strain showed a 6.4-fold increase in TET resistance (Supplementary Data 2). Contributions of *acrR* and *ompF* mutations to TET resistance are described in the main text. Two TET evolved strains (TETE2 and TETE4) had mutations in *rpoB* encoding the β subunit of RNA polymerase and a reconstructed *rpoB* mutant strain carrying the *rpoB* G713C mutation, which was found in the TET4 strain showed a 1.9-fold increase in TET resistance (Supplementary Data 2). In addition to the contribution of the *rpoB* mutation to RFP resistance [59], mutations in *rpoB* also confer antibiotic resistance e.g. a fluoroquinolone antibiotic ciprofloxacin (CPFX) [60]. It was shown that *rpoB* mutations conferring resistance to CPFX were located outside of the RFP resistance-determining region (RRDR) of *rpoB* and these mutations resulted in increased expression of *mdtK* encoding the multidrug efflux transporter [60]. However, TETE2 and TET4 strains did not show increased *mdtK* expression (Supplementary Data 3). These results suggest that *rpoB* mutations also affect the transcriptome, beyond *mdtK*-induced resistance to TET. Two TET evolved strains (TETE4 and TETE6) had mutations in *rob* encoding a transcriptional dual regulator; a reconstructed *rob* mutant strain carrying *rob* R156H mutation, which was found in TETE4, showed an 8.9-fold increase in TET resistance (Supplementary Data 2). MarR, SoxR, and Rob share a common regulon core including genes related to multidrug resistance e.g. *acrAB* and *ompF* [61]. A previous study also reported that mutations in *rob* resulted in CP, TET, CPFX, and piperacillin resistance [61]. In this study, we also confirmed that the rob R156H mutation resulted in cross-resistance to several drugs including CP, CMZ, AZT, CBPC, NFLX, TET, EM, and PUR, while this mutation also resulted in collateral sensitivity to LVAL and B-Cl-Ala (Supplementary Data 2).

VCM evolved strains

Vancomycin hydrochloride (VCM) binds to D-Ala-D-Ala resulting in inhibition of peptidoglycan synthesis. Two VCM evolved strains had point mutations in *dacA* (G359R in VCME2 and F219L in VCME5) encoding D-alanyl-D-alanine carboxypeptidase PBP5 and a reconstructed *dacA* mutant strain carrying G359R mutation showed a 1.6-fold increase in VCM resistance. It was reported that PBP5 is involved in resistance to β-lactam and vancomycin in Enterococci [62]. These results suggest that the *dacA* mutations affect transpeptidation reactions resulting in VCM resistance in *E. coli*.

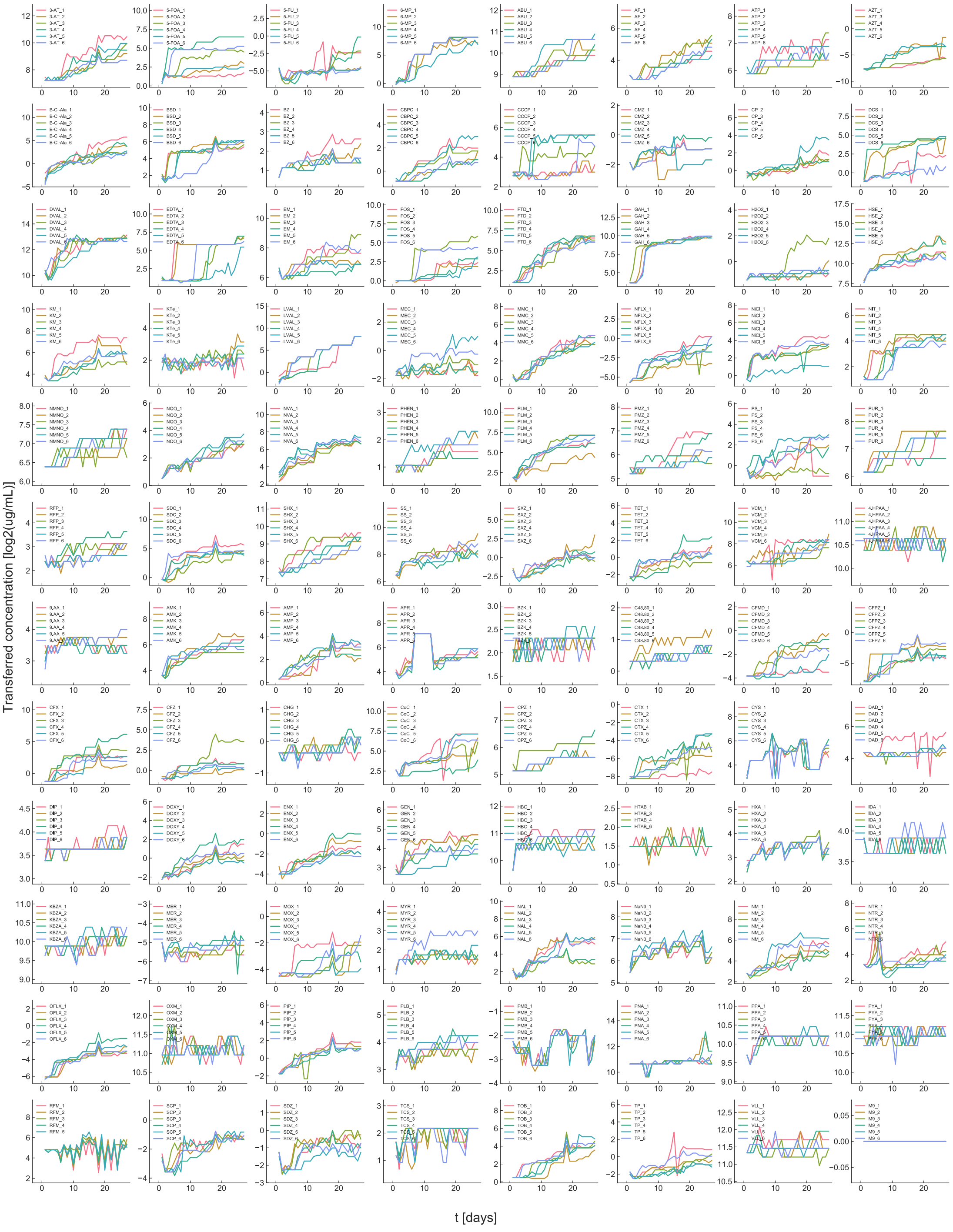

Supplementary Fig. 1. Resistance time series for laboratory evolution

Time series of the highest drug concentration in which the cells could grow (transferred concentration) during the laboratory evolution experiment. The drugs are sorted based on the order of Supplementary Table 1. Source data are provided as a Source Data file.

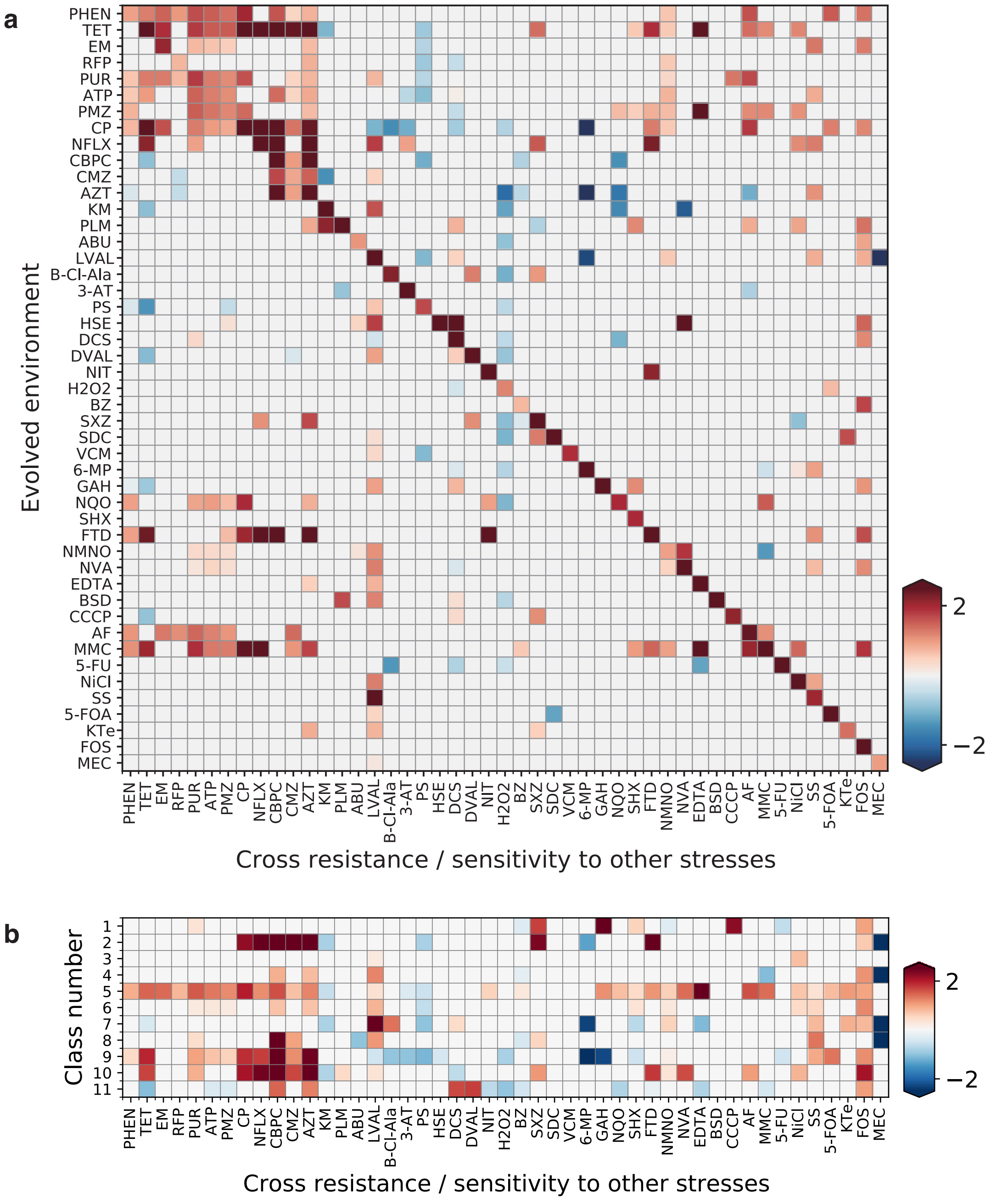

Supplementary Fig. 2. Combinations of stresses which exhibited cross resistance and collateral sensitivity

a, Identified combinations of stresses that exhibited either cross resistance or collateral sensitivity for each of the four strains which evolved in the same environment. **b,** Combinations of stresses which exhibited either cross resistance or collateral sensitivity for the strains in each class in the supervised principal component analysis (PCA) space. The combinations were detected by the Mann-Whitney U-test (two-sided, false discovery rate, FDR < 0.05), and the colors indicate the resistance to the stress relative to the parent strain. Source data are provided as a Source Data file.

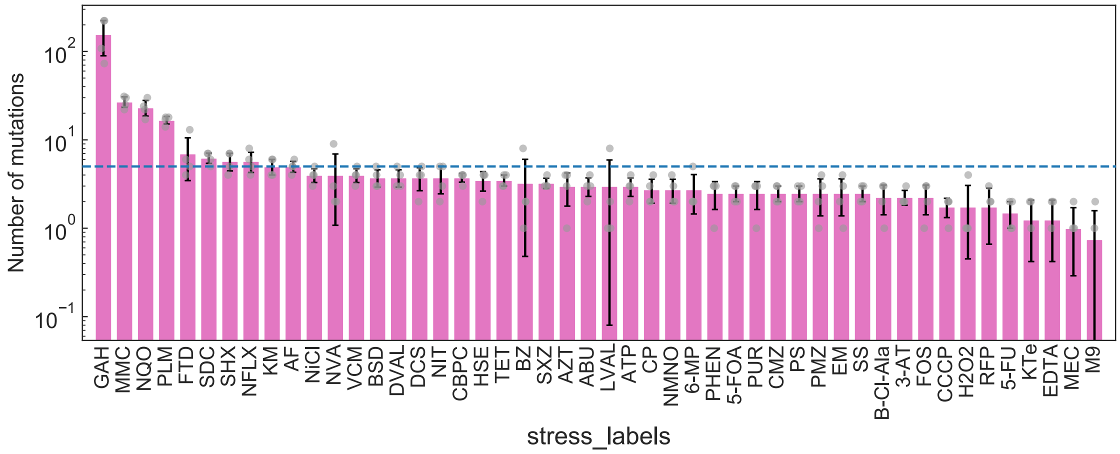

Supplementary Fig. 3. Number of mutations identified in the evolved strains. The mean number of identified mutations for the four evolved strains in each environment are shown. Error bars are presented as mean values +/- standard deviation calculated from n=4 biologically independent culture lines. Source data are provided as a Source Data file.

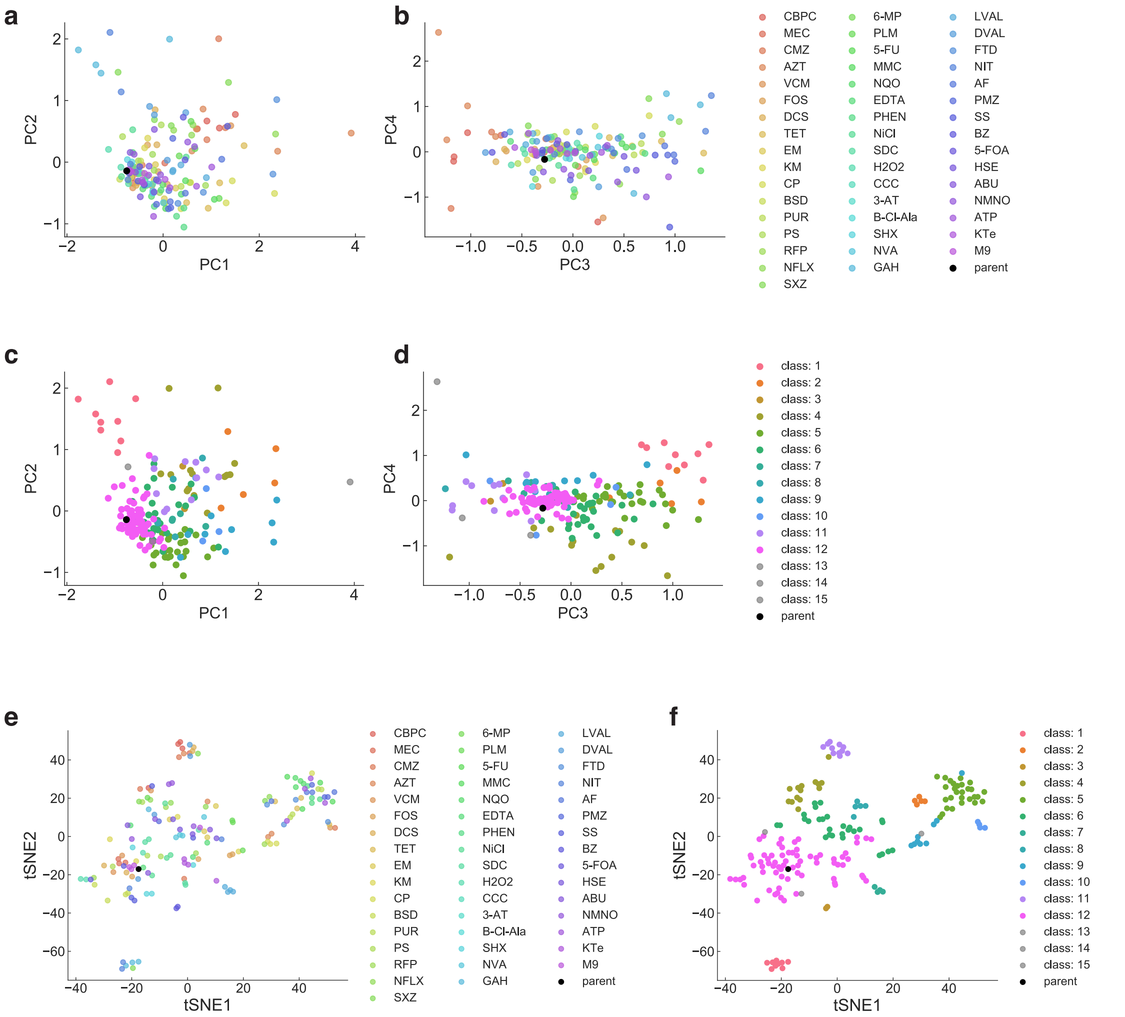

Supplementary Fig. 4. PCA and tSNE plot of the supervised PCA space

a, b, Distribution of the 192 evolved strains and the parent strain in the supervised PCA space. The four principal components are shown and the colors denote the evolved environment for each strain. **c, d,** Data is the same as in a,b is plotted. The colors denote the class defined by hierarchical clustering. **e,** The tSNE plot for the distribution of the evolved strains in the 36 dimension supervised PCA space. Colors denote the evolved environment. **f,** Data is the same as in e. Colors denote the classes defined by hierarchical clustering. Source data are provided as a Source Data file.

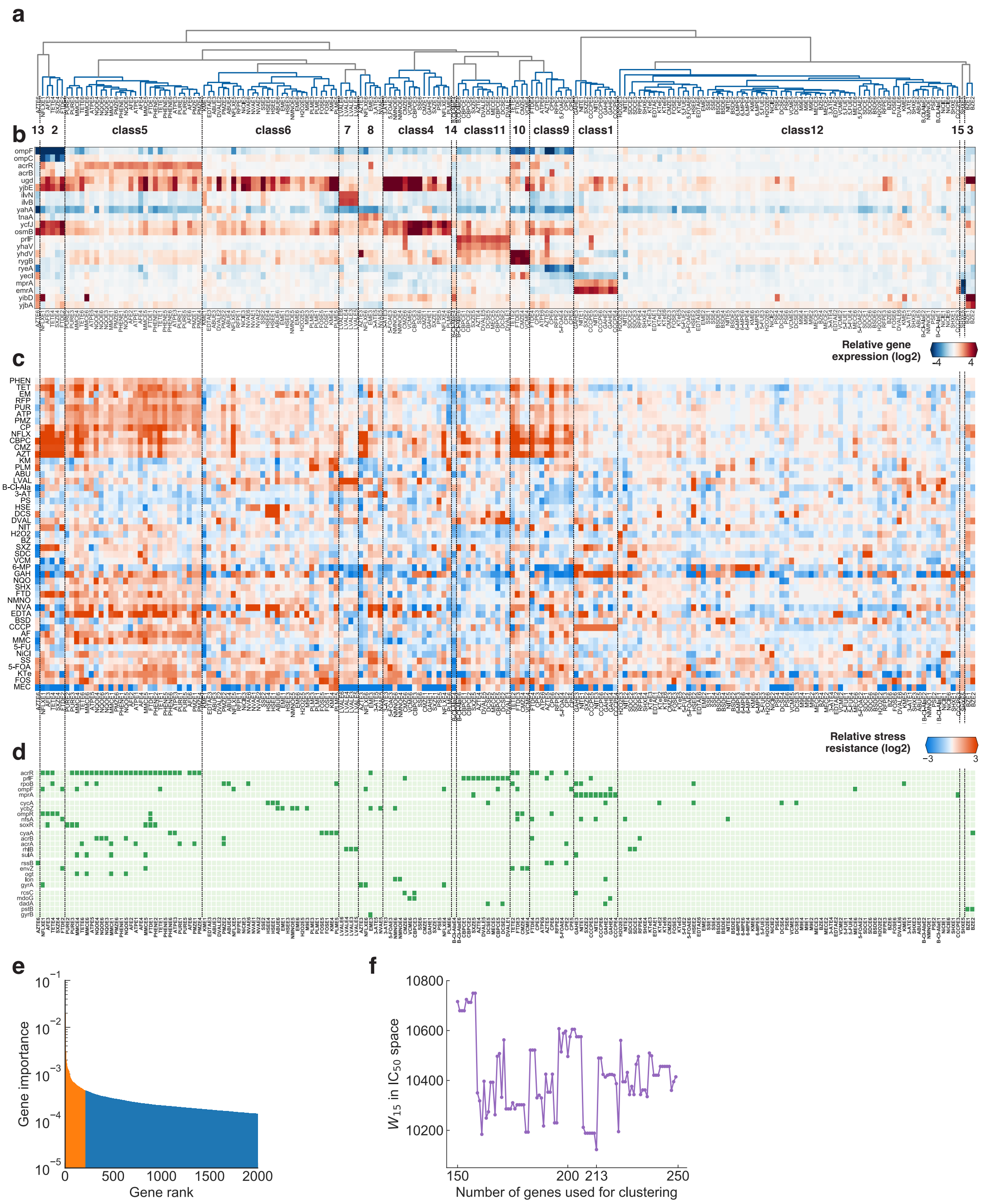

Supplementary Fig. 5. Phenotypic and genotypic characteristics for all 15 supervised PCA classes

a, Dendrogram of the result of hierarchical clustering performed in the 36 dimensional supervised PCA space. **b,** Gene expression levels of representative genes for each cluster, relative to the parent strain. The genes were selected from the intersection of the top two gene weights for the linear discriminant analysis (LDA) axis and differentially expressed genes (STAR Methods). **c,** Half-maximal inhibitory concentrations (IC_{50}) values relative

to the parent strain. **d,** Commonly mutated genes within the evolved strains. Mutated genes enriched for each cluster clarified by Fisher's exact test (two-sided, $p < 0.01$) are presented. The exact p-values for the enriched genes are as follows: *acrR* ($p=9\times10^{-22}$), *prlF* ($p=7\times10^{-13}$), *mprA* ($p=8\times10^{-4}$), *mprA* ($p=8\times10^{-4}$), *ycbZ* ($p=2\times10^{-3}$), *ompR* ($p=6\times10^{-6}$), *soxR* ($p=4\times10^{-5}$), *cyaA* ($p=9\times10^{-3}$), *acrB* ($p=9\times10^{-3}$), *acrA* ($p=8\times10^{-4}$), *rhlB* ($p=7\times10^{-5}$), *sulA* ($p=4\times10^{-4}$), *rssB* ($p=6\times10^{-4}$), *envZ* ($p=3\times10^{-5}$), *gyrA* ($p=3\times10^{-3}$), *rcsC* ($p=4\times10^{-3}$), *ogt* ($p=3\times10^{-3}$), *mdoG* ($p=5\times10^{-3}$), *dadA* ($p=3\times10^{-3}$), *pstB* ($p=5\times10^{-5}$). Mutated genes that were identified in more than seven strains are also presented. **e,** Feature importance of the genes computed by the random forest regression model. **f,** Class dissimilarity W_{15} for the results of hierarchical clustering in the supervised PCA spaces constructed by different number of genes. The genes are sorted in the order of gene importance given by the random forest model. Source data are provided as a Source Data file.

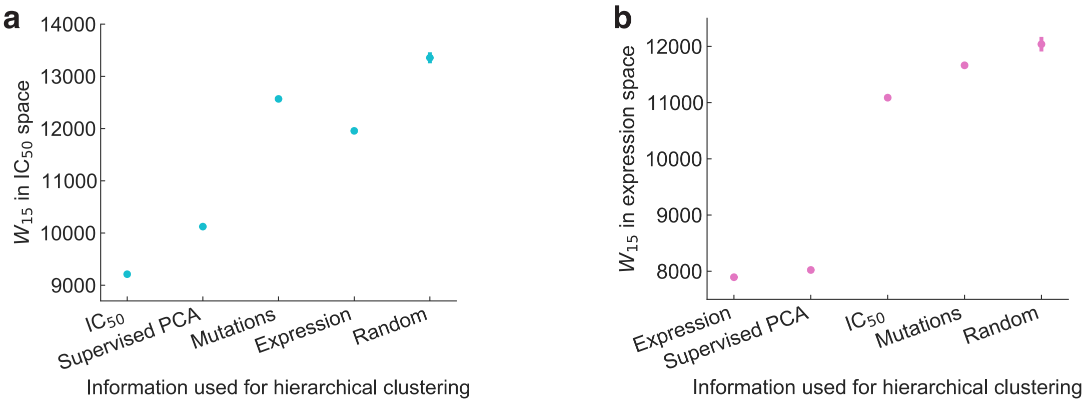

Supplementary Fig. 6. Accessing the validity of clustering in the supervised PCA space

a, Class dissimilarity W_{15} in the IC_{50} space for the 15 classes which were defined by hierarchical clustering based on the supervised PCA expression space, IC_{50} space, mutations, and full gene expression space, respectively, are shown. The mean and standard deviation for the class dissimilarity for ten runs of randomly clustered results in the IC_{50} space are also shown. **b,** Class dissimilarity W_{15} in the 4492 dim. gene expression space for the results of hierarchical clustering in other spaces are shown. The mean and standard deviation for the class dissimilarity for ten runs of randomly clustered results in the gene expression space are also shown. Source data are provided as a Source Data file.

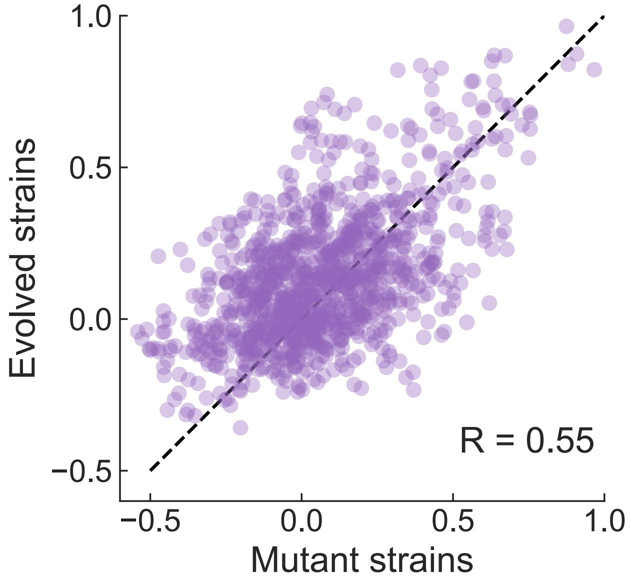

Supplementary Fig. 7. Phenotypic correspondence of the evolved strains and mutant strains without transporter related mutations

Relationships between the corresponding pairwise correlation coefficients for the evolved strains and the mutant strains. Here, transporter related mutations (*dctA, uraA, sstT, livM, potA, oppA, cycA, yhjE, glpT, ompF, glnP, metN, ptsP, frlA, gabP, potH, mprA,* and *acrR*) were excluded from the mutant strains. Source data are provided as a Source Data file.

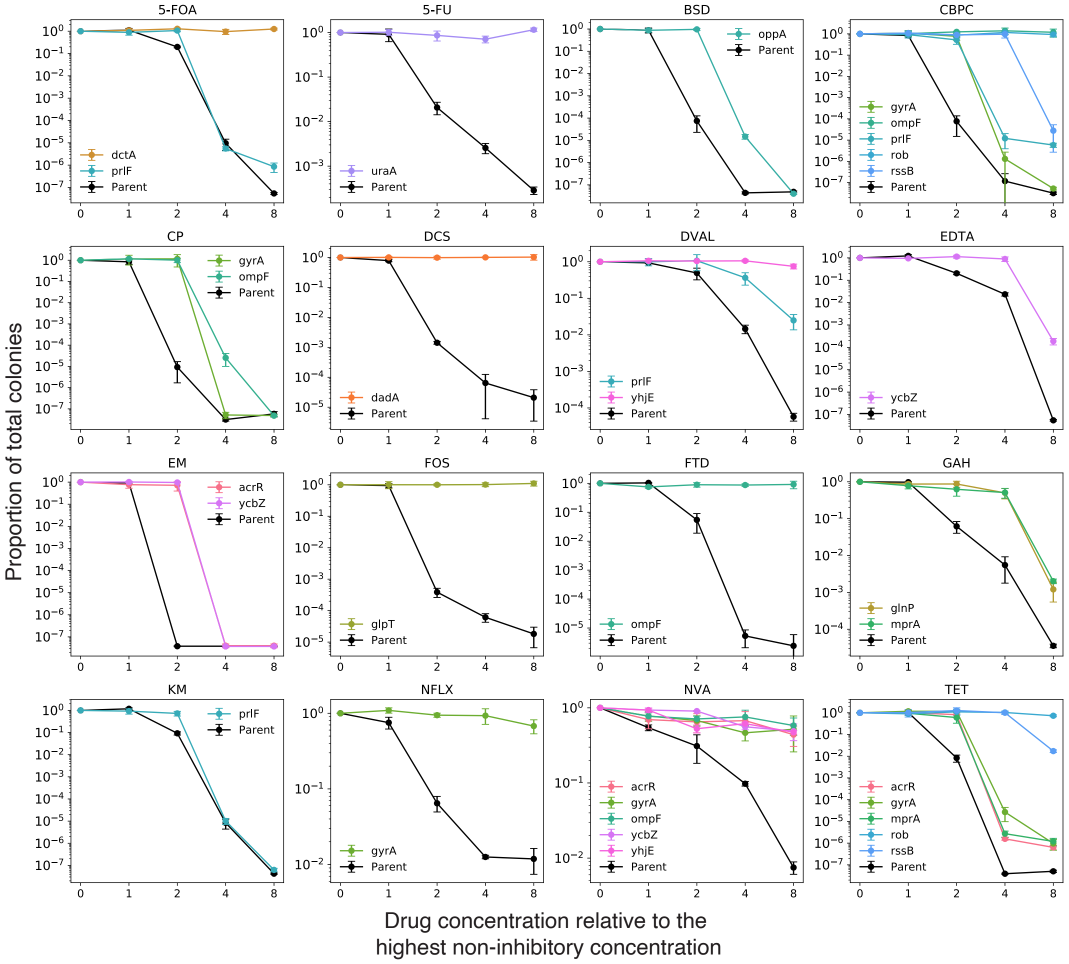

Supplementary Fig. 8. Both genetic resistant and heteroresistant strains were observed within the mutant strains.

Colony-forming units of the parent strain and mutant strains for increasing concentrations of a respective drug. The CFU for each strain is normalized by the CFU of the no drug condition. Here, the highest non-inhibitory concentration, represented by the concentration where the CFU of the parent strain is 50% less than that of the no drug condition, is set to 1. Data are presented as mean values +/- standard deviation for n=3 biologically independent samples. Source data are provided as a Source Data file.

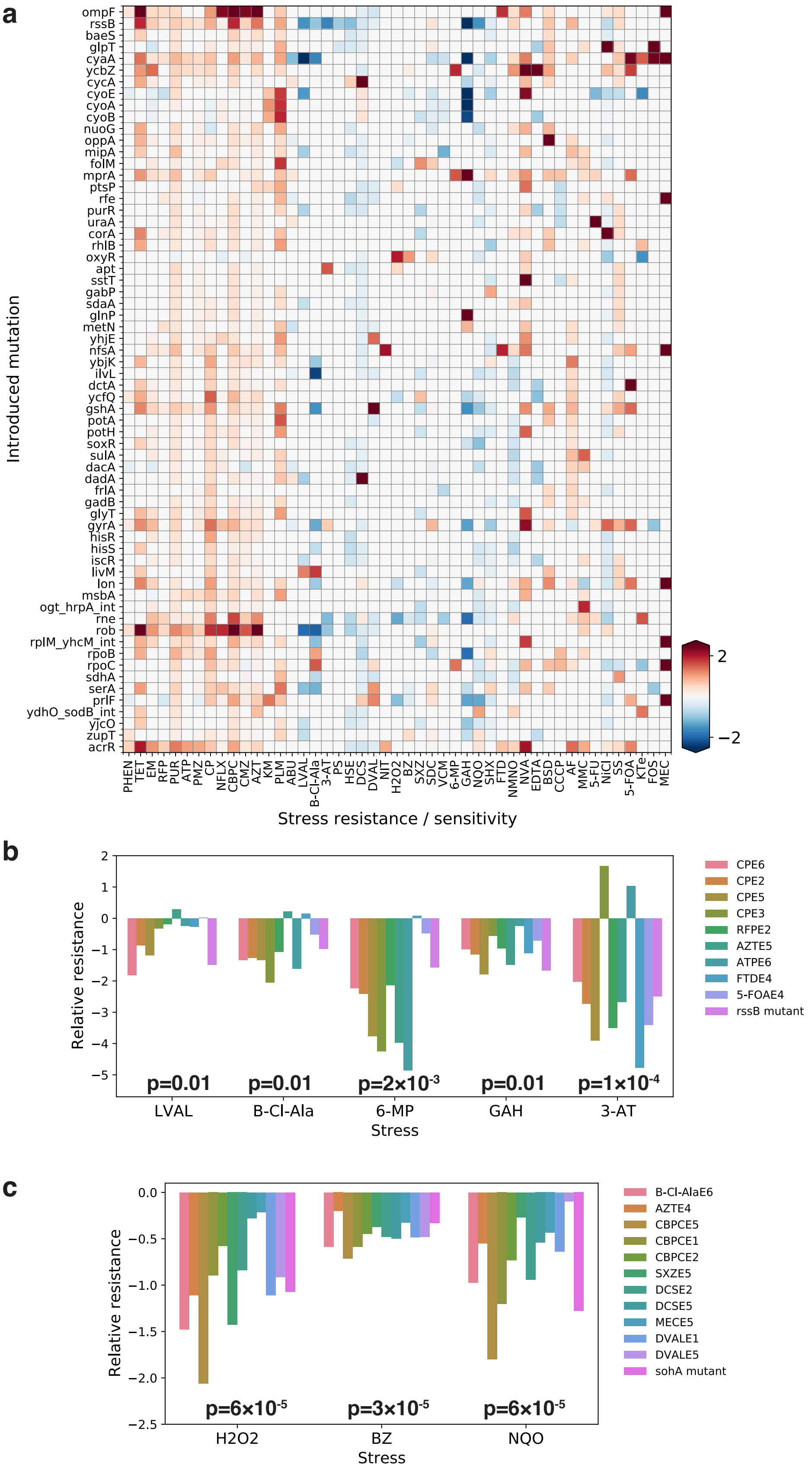

a
ompF
rssB
baeS
glpT
cyaA
ycbZ
cycA
cyoE
cyoA
cyoB
nuoG
oppA
mipA
folM
mprA
ptsP
rfe
purR
uraA
corA
rhlB
oxyR
apt
sstT
gabP
sdaA
glnP
metN
yhjE
nfsA
ybjK
ilvL
dctA
ycfQ
gshA
potA
potH
soxR
sulA
dacA
dadA
frlA
gadB
glyT
gyrA
hisR
hisS
iscR
livM
lon
msbA
ogt_hrpA_int
rne
rob
rplM_yhcM_int
rpoB
rpoC
sdhA
serA
prlF
ydhO_sodB_int
yjcO
zupT
acrR
Introduced mutation
PHEN
TET
EM
RFP
PUR
ATP
PMZ
CP
NFLX
CBPC
CMZ
AZT
KM
PLM
ABU
LVAL
B-Cl-Ala
3-AT
PS
HSE
DCS
DVAL
NIT
H2O2
BZ
SXZ
SDC
VCM
6-MP
GAH
NQO
SHX
FTD
NMNO
NVA
EDTA
BSD
CCCP
AF
MMC
5-FU
NiCl
SS
5-FOA
KTe
FOS
MEC
Stress resistance / sensitivity
2
−2
b
Relative resistance
p=0.01
p=0.01
p=2×10-3
p=0.01
p=1×10-4
LVAL
B-Cl-Ala
6-MP
GAH
3-AT
Stress
CPE6
CPE2
CPE5
CPE3
RFPE2
AZTE5
ATPE6
FTDE4
5-FOAE4
rssB mutant
c
Relative resistance
p=6×10-5
p=3×10-5
p=6×10-5
H2O2
BZ
NQO
Stress
B-Cl-AlaE6
AZTE4
CBPCE5
CBPCE1
CBPCE2
SXZE5
DCSE2
DCSE5
MECE5
DVALE1
DVALE5
sohA mutant

Supplementary Fig. 9. Combinations of stresses which exhibited cross resistance, collateral sensitivity

a, Stresses which exhibited either resistance or sensitivity for the 64 reconstructed mutant strains (Mann-Whitney U-test, two-sided, $p < 0.05$). The exact p-values are provided in source data. Colors indicate the stress resistance relative to the parent strain. **b, c,** Stress resistance relative to the parent strain for strains in class 9 and class 11, respectively. Resistance levels for the *rssB* and *prlF* mutant are also shown for comparison. The p-values were calculated by the Mann-Whitney U-test (two-sided), which compared the evolved strains in the corresponding class and the parent strains. Source data are provided as a Source Data file.

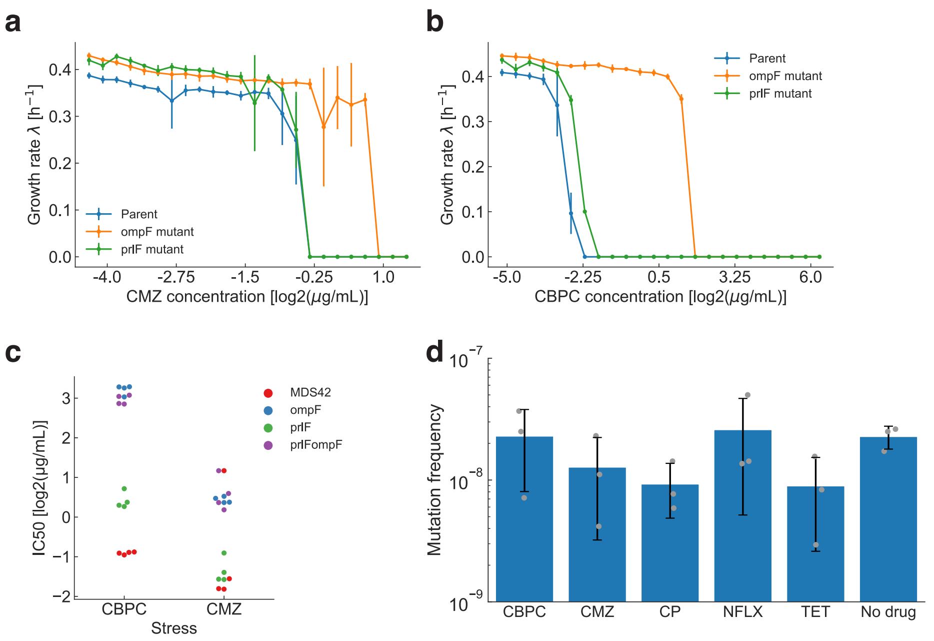

Supplementary Fig. 10. Neither fitness trade-offs nor epistasis nor mutation frequency explains decelerated evolution

a, b, Growth rates for the parent strain, *ompF* mutated strain, and the *prlF* mutant. Growth rates were measured in 24 concentration levels of cefmetazole (CMZ) and carbenicillin (CBPC), respectively. **c,** IC_{50} levels measured for different stresses for the parent strain, *ompF* mutated strain, *prlF* mutated strain, and the *prlF*/*ompF* doubled mutated strain, respectively**. d,** Mutation frequencies for MDS42 strains under addition of IC_{50} concentrations of β-lactam stresses (i.e. CBPC, CMZ) and other antibiotics in which the evolved strains acquired high resistance to β-lactam stresses (i.e. CP, NFLX, TET). Error bars are presented as mean values +/- standard deviation of three independent experiments. Source data are provided as a Source Data file.

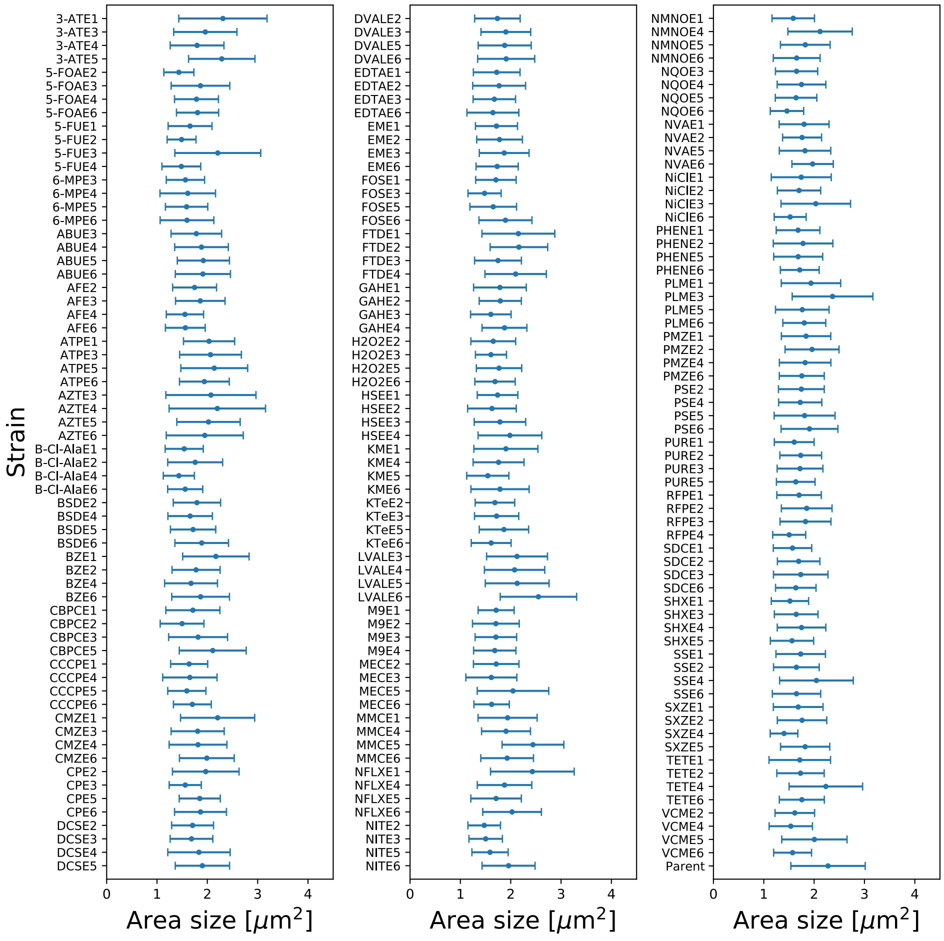

Supplementary Fig. 11. Area sizes of all 192 evolved and parent strains.
Mean area sizes for single cell observations of the evolved strains and parent strain are shown. Data are presented as mean values +/- standard deviation for n≈158 cells in average for each strain. Source data are provided as a Source Data file.

Supplementary References

1. Bond, T. J. & Akers, J. Mechanism of growth inhibition of *Escherichia coli* by 3-amino-1,2,4-triazole. *J. Bacteriol.* **81**, 327–328 (1961).
2. Meng, X., Smith, R. M., Giesecke, A. V., Joung, J. K. & Wolfe, S. A. Counter-selectable marker for bacterial-based interaction trap systems. *Biotechniques* **40**, 179–184 (2006).
3. Levine, R. A. & Taylor, M. W. Selection for purine regulatory mutants in an *E. coli* hypoxanthine phosphoribosyl transferase-guanine phosphoribosyl transferase double mutant. *MGG Mol. Gen. Genet.* **181**, 313–318 (1981).
4. Kumar, S. & Doerrler, W. T. Members of the conserved DedA family are likely membrane transporters and are required for drug resistance in *Escherichia coli*. *Antimicrob. Agents Chemother.* **58**, 923–930 (2014).
5. Shimada, T. *et al.* A novel regulator RcdA of the *csgD* gene encoding the master regulator of biofilm formation in *Escherichia coli*. *Microbiologyopen* **1**, 381–394 (2012).
6. Georgopapadakou, N. H., Smith, S. A. & Sykes, R. B. Mode of action of azthreonam. *Antimicrob. Agents Chemother.* **21**, 950–956 (1982).
7. Nagakubo, S., Nishino, K., Hirata, T. & Yamaguchi, A. The putative response regulator BaeR stimulates multidrug resistance of *Escherichia coli* via a novel multidrug exporter system , MdtABC. *J. Bacteriol.* **184**, 4161–4167 (2002).
8. Hirakawa, H., Nishino, K., Hirata, T. & Yamaguchi, A. Comprehensive studies of drug resistance mediated by overexpression of response regulators of two-component signal transduction systems in *Escherichia coli*. *J. Bacteriol.* **185**, 1851–1856 (2003).
9. Miller-Fleming, L., Olin-Sandoval, V., Campbell, K. & Ralser, M. Remaining mysteries of molecular biology : The role of polyamines in the cell. *J. Mol. Biol.* **427**, 3389–3406 (2015).
10. Piddock, L. J. V. & Wise, R. Properties of the penicillin-binding proteins of four species of the genus *Bacteroides*. *Antimicrob. Agents Chemother.* **29**, 825–832 (1986).
11. Lomovskaya, O., Lewis, K. & Matin, A. EmrR is a negative regulator of the *Escherichia coli* multidrug resistance pump EmrAB. *J. Bacteriol.* **177**, 2328–2334 (1995).
12. Ishino, F. *et al.* Peptidoglycan synthetic activities in membranes of *Escherichia coli* caused by overproduction of penicillin-binding protein 2 and RodA protein. *J. Biol. Chem.* **261**, 7024–7031 (1986).
13. Fehér, T., Cseh, B., Umenhoffer, K., Karcagi, I. & Pósfai, G. Characterization of *cycA* mutants of *Escherichia coli*. An assay for measuring *in vivo* mutation rates. *Mutat. Res.* **595**, 184–190 (2006).
14. Baisa, G., Stabo, N. J. & Welch, R. A. Characterization of *Escherichia coli* D-cycloserine transport and resistant mutants. *J. Bacteriol.* **195**, 1389–1399 (2013).
15. Qi, H. *et al.* Effects of D-valine on periodontal or peri-implant pathogens: *Porphyromonas gingivalis* biofilm. *J. Periodontol.* **89**, 303–314 (2018).

16. Greenberg, J. T. & Demple, B. Glutathione in *Escherichia coli* is dispensable for resistance to H202 and gamma radiation. *J. Bacteriol.* **168**, 1026–1029 (1986).
17. Harrison, J. J. *et al.* Chromosomal antioxidant genes have metal ion-specific roles as determinants of bacterial metal tolerance. *Environ. Microbiol.* **11**, 2491–2509 (2009).
18. Modi, S. R., Camacho, D. M., Kohanski, M. A., Walker, G. C. & Collins, J. J. Functional characterization of bacterial sRNAs using a network biology approach. *Proc. Natl. Acad. Sci.* **108**, 15522–15527 (2011).
19. Gray, B. G. W. & Wilkinson, S. G. The effect of ethylendiaminetetra-acetic acid on the cell walls of some Gram-negative bacteria. *J. Gen. Microbiol.* **39**, 385–399 (1965).
20. Santoro, A. *et al.* Interaction of fosfomycin with the Glycerol 3- phosphate transporter of *Escherichia coli*. *Biochim. Biophys. Acta* **1810**, 1323–1329 (2011).
21. Martín-Gutiérrez, G. *et al.* Urinary tract conditions affect fosfomycin activity against *Escherichia coli* strains harboring chromosomal mutations involved in fosfomycin uptake. *Antimicrob. Agents Chemother.* **62**, e01899-17 (2017).
22. Mccalla, D. R., Kaiser, C. & Green, M. H. L. Genetics of nitrofurazone resistance in *Escherichia coli*. *J. Bacteriol.* **133**, 10–16 (1978).
23. Whiteway, J. *et al.* Oxygen-insensitive nitroreductases : analysis of the roles of *nfsA* and *nfsB* in development of resistance to 5-nitrofuran derivatives in *Escherichia coli*. *J. Bacteriol.* **180**, 5529–5539 (1998).
24. Christman, M. F., Storz, G. & Ames, B. N. OxyR, a positive regulator of hydrogen peroxide-inducible genes in *Escherichia coli* and *Salmonella typhimurium*, is homologous to a family of bacterial regulatory proteins. *Proc. Natl. Acad. Sci.* **86**, 3484–3488 (1989).
25. Kotre, A. M., Sullivan, S. J. & Savageau, M. A. Metabolic regulation by homoserine in *Escherichia coli* B/r. *J. Bacteriol.* **116**, 663–672 (1973).
26. Suzuki, S., Horinouchi, T. & Furusawa, C. Prediction of antibiotic resistance by gene expression profiles. *Nat. Commun.* **5**, 5792 (2014).
27. Mogre, A., Sengupta, T., Veetil, R. T., Ravi, P. & Seshasayee, A. S. N. Genomic analysis reveals distinct concentration-dependent evolutionary trajectories for antibiotic resistance in *Escherichia coli*. *DNA Res.* **21**, 711–726 (2014).
28. Mogre, A., Veetil, R. T. & Seshasayee, A. S. N. Modulation of global transcriptional regulatory networks as a strategy for increasing kanamycin resistance of the translational elongation factor-G mutants in *Escherichia coli*. *G3 Genes Genomes Genet.* **7**, 3955–3966 (2017).
29. Ezraty, B. *et al.* Fe-S cluster biosynthesis controls uptake of aminoglycosides in a ROS-less death pathway. *Science* **340**, 1583–1588 (2013).
30. Acosta, M. B. R., Ferreira, R. C. C., Padilla, G., Ferreira, L. C. S. & Costa, S. O. P. Altered expression of oligopeptide-binding protein (OppA) and aminoglycoside resistance in laboratory and clinical *Escherichia coli* strains. *J. Med. Microbiol.* **49**, 409–413 (2000).
31. Nakamatsu, E. H. *et al.* Oligopeptide uptake and aminoglycoside resistance in

Escherichia coli K12. *FEMS Microbiol. Lett.* **269**, 229–233 (2007).
32. Pérez, J. M. *et al.* Bacterial toxicity of potassium tellurite: Unveiling an ancient enigma. *PLoS One* **2**, e211 (2007).
33. Vanzo, N. F. *et al.* Ribonuclease E organizes the protein interactions in the *Escherichia coli* RNA degradosome. *Genes Dev.* **12**, 2770–2781 (1998).
34. Mackie, G. A. RNase E: At the interface of bacterial RNA processing and decay. *Nat. Rev. Microbiol.* **11**, 45–57 (2013).
35. Favre, R. *et al.* Expression of a valine-resistant acetolaetate synthase activity mediated by the *ilrO* and *ilvG* genes of *Escherichia coli* K-12. *Mol. Gen. Genet. MGG* **252**, 243–252 (1976).
36. Blatt, J. M., Pledgner, W. J. & Umbarger, H. E. Isoleucine and valine metabolism in *Escherichia coli*. XX. Multiple forms of acetohydroxy acid synthetase. *Biochem. Biophys. Res. Commun.* **48**, 444–450 (1972).
37. Leavitt, R. I. & Umbarger, H. E. Isoleucine and valine metabolism in *Escherichia coli*. XI. Valine inhibition of the growth of Escherichia coli strain K-12. *J. Bacteriol.* **83**, 624–630 (1962).
38. Ogura, T. *et al.* Penicillin-binding protein 2 is essential in wild-type *Escherichia coli* but not in *lov* or *cya* mutants. *J. Bacteriol.* **171**, 3025–3030 (1989).
39. Rebeck, G. W. & Samson, L. Increased spontaneous mutation and alkylation sensitivity of *Escherichia coli* strains lacking the *ogt* 06-methylguanine DNA repair methyltransferase. *J. Bacteriol.* **173**, 2068–2076 (1991).
40. Zhang, D., Li, H., Lin, X. & Peng, X. Outer membrane proteomics of kanamycin-resistant *Escherichia coli* identified MipA as a novel antibiotic resistance-related protein. *FEMS Microbiol. Lett.* **362**, fnv074 (2015).
41. Nicoloff, H. & Andersson, D. I. Lon protease inactivation, or translocation of the *lon* gene, potentiate bacterial evolution to antibiotic resistance. *Mol. Microbiol.* **90**, 1233–1248 (2013).
42. Downes, D. J. *et al.* Characterization of the mutagenic spectrum of 4-nitroquinoline 1-oxide (4-NQO) in Aspergillus nidulans by whole genome sequencing. *G3 Genes Genomes Genet.* **4**, 2483–2492 (2014).
43. Keyer, K., Gort, A. S. & Imlay, J. A. Superoxide and the production of oxidative DNA damage. *J. Bacteriol.* **177**, 6782–6790 (1995).
44. Moore, J. M., Correa, R., Rosenberg, S. M. & Hastings, P. J. Persistent damaged bases in DNA allow mutagenic break repair in *Escherichia coli*. *PLoS Genet.* **13**, e1006733 (2017).
45. Kikuchi, Y., Kojima, H. & Tanaka, T. Mutational analysis of the feedback sites of lysine-sensitive aspartokinase of *Escherichia coli*. *FEMS Microbiol. Lett.* **173**, 211–215 (1999).
46. Schwartz, C. J. *et al.* IscR, an Fe-S cluster-containing transcription factor, represses expression of *Escherichia coli* genes encoding Fe-S cluster assembly proteins. *Proc. Natl. Acad. Sci.* **98**, 14895–14900 (2001).
47. Amaral, L. & Viveiros, M. Thioridazine: A non-antibiotic drug highly effective, in combination with first line anti-tuberculosis drugs, against any form of

antibiotic resistance of *Mycobacterium tuberculosis* due to its multi-mechanisms of action. *Antibiotics* **6**, E3 (2017).
48. Aspedon, A. & Groisman, E. A. The antibacterial action of protamine: evidence for disruption of cytoplasmic membrane energ ization in *Salmonella typhimurium*. *Microbiology* **142**, 3389–3397 (1996).
49. Hobbs, E. C., Yin, X., Paul, B. J., Astarita, J. L. & Storz, G. Conserved small protein associates with the multidrug efflux pump AcrB and differentially affects antibiotic resistance. *Proc. Natl. Acad. Sci.* **109**, 16696–16701 (2012).
50. Vinué, L., Corcoran, M. A., Hooper, D. C. & Jacoby, G. A. Mutations that enhance the ciprofloxacin resistance of *Escherichia coli* with *qnrA1*. *Antimicrob. Agents Chemother.* **60**, 1537–1545 (2016).
51. Okusu, H., Ma, D. & Nikaido, H. AcrAB efflux pump plays a major role in the antibiotic resistance phenotype of *Escherichia coli* multiple- antibiotic-resistance (Mar) mutants. *J. Bacteriol.* **178**, 306–308 (1996).
52. Ackerley, D. F., Barak, Y., Lynch, S. V, Curtin, J. & Matin, A. Effect of chromate stress on *Escherichia coli* K-12. *J. Bacteriol.* **188**, 3371–3381 (2006).
53. Mckitrickt, J. C. & Pizert, L. I. Regulation of phosphoglycerate dehydrogenase levels and effect on serine synthesis in *Escherichia coli* K-12. *J. Bacteriol.* **141**, 235–245 (1980).
54. Domenico, P., Schwartz, S. & Cunha, B. A. Reduction of capsular polysaccharide production in *Klebsiella pneumoniae* by sodium salicylate. *Infect. Immun.* **57**, 3778–3782 (1989).
55. Kunin, C. M., Hua, T. H. & Bakaletz, L. O. Effect of salicylate on expression of flagella by *Escherichia coli* and *Proteus*, *Providencia*, and *Pseudomonas* spp. *Infect. Immun.* **63**, 1796–1799 (1995).
56. Vila, J. & Soto, S. M. Salicylate increases the expression of *marA* and reduces in vitro biofilm formation in uropathogenic *Escherichia coli* by decreasing type 1 fimbriae expression. *Virulence* **3**, 280–285 (2012).
57. Belt, K. *et al.* Salicylic acid-dependent plant stress signaling via mitochondrial succinate dehydrogenase. *Plant Physiol.* **173**, 2029–2040 (2017).
58. Girgis, H. S., Hottes, A. K. & Tavazoie, S. Genetic architecture of intrinsic antibiotic susceptibility. *PLoS One* **4**, e5629 (2009).
59. Jin, D. J. & Gross, C. A. Mapping and sequencing of mutations in the *Escherichia coli rpoB* gene that lead to rifampicin resistance. *J. Mol. Biol.* **202**, 45–58 (1988).
60. Pietsch, F. *et al.* Ciprofloxacin selects for RNA polymerase mutations with pleiotropic antibiotic resistance effects. *J. Antimicrob. Chemother.* **72**, 75–84 (2017).
61. Munck, C., Gumpert, H. K., Wallin, A. I. N., Wang, H. H. & Sommer, M. O. A. Prediction of resistance development against drug combinations by collateral responses to component drugs. *Sci. Transl. Med.* **6**, 262ra156 (2014).
62. Arthura, M. & Courvalin, P. Genetics and mechanisms of glycopeptide resistance in *Enterococci*. *Antimicrob. Agents Chemother.* **37**, 1563–1571 (1993).
