# Supplementary material for: High-throughput laboratory evolution reveals evolutionary constraints in Escherichia coli
Source: Nat Commun. 2020 Nov 24;11:5970. doi: 10.1038/s41467-020-19713-w (PMC7686311; doi:10.1038/s41467-020-19713-w)
Supplement: Supplementary file 3 — Description of Additional Supplementary Files [file 41467_2020_19713_MOESM3_ESM.pdf]

Description of Additional Supplementary Files

File Name: Supplementary Data 1
Description: List of the chemicals used in this study

File Name: Supplementary Data 2
Description: All relative half-maximal inhibitory concentrations (IC50s) for the 47 stresses of each evolved strain and reconstructed mutant strain

File Name: Supplementary Data 3
Description: All identified mutations in the evolved strains.
All identified mutations in the evolved strains are shown in sheet 1. Representative genes in which non-synonymous mutations or ins/dels were commonly fixed in the evolved strains are listed in sheet 2. Primers used for the constructions of the reconstructed mutant strains are also shown in sheet 2.

File Name: Supplementary Data 4
Description: Transcriptome data of evolved strains.

File Name: Supplementary Data 5
Description: Cross-resistances and collateral sensitivities observed in the reconstructed mutant strains. In addition to the 64 mutant strains that were mentioned in the main text, two mutant strains that could not grow in M9 (*cysP, hpt*) and the *fusA* strain which did not grow under several stresses are included here. The *cysP, hpt* strains were grown in the M9 + 20 amino acids medium. Chemicals that were identified as significantly increased or decreased IC50 values (Mann-Whitney U-test, false discovery rate (FDR) < 5%) in the all reconstructed mutant strains are shown.
