# Supplementary material for: High-throughput laboratory evolution reveals evolutionary constraints in Escherichia coli
Source: Nat Commun. 2020 Nov 24;11:5970. doi: 10.1038/s41467-020-19713-w (PMC7686311; doi:10.1038/s41467-020-19713-w)
Supplement: Supplementary file 9 — Reporting Summary [file 41467_2020_19713_MOESM9_ESM.pdf]

nature research

Corresponding author(s): Tomoya Maeda, Chikara Furusawa
Last updated by author(s): Oct 14, 2020

Reporting Summary

Nature Research wishes to improve the reproducibility of the work that we publish. This form provides structure for consistency and transparency in reporting. For further information on Nature Research policies, see Authors & Referees and the Editorial Policy Checklist .

Statistics

For all statistical analyses, confirm that the following items are present in the figure legend, table legend, main text, or Methods section.

n/a	Confirmed	
☐	☒	The exact sample size (n) for each experimental group/condition, given as a discrete number and unit of measurement
☐	☒	A statement on whether measurements were taken from distinct samples or whether the same sample was measured repeatedly
☐	☒	The statistical test(s) used AND whether they are one- or two-sided<br>*Only common tests should be described solely by name; describe more complex techniques in the Methods section.*
☐	☒	A description of all covariates tested
☐	☒	A description of any assumptions or corrections, such as tests of normality and adjustment for multiple comparisons
☐	☒	A full description of the statistical parameters including central tendency (e.g. means) or other basic estimates (e.g. regression coefficient) AND variation (e.g. standard deviation) or associated estimates of uncertainty (e.g. confidence intervals)
☐	☒	For null hypothesis testing, the test statistic (e.g. F, t, r) with confidence intervals, effect sizes, degrees of freedom and P value noted<br>*Give P values as exact values whenever suitable.*
☒	☐	For Bayesian analysis, information on the choice of priors and Markov chain Monte Carlo settings
☒	☐	For hierarchical and complex designs, identification of the appropriate level for tests and full reporting of outcomes
☐	☒	Estimates of effect sizes (e.g. Cohen's d, Pearson's r), indicating how they were calculated

Our web collection on statistics for biologists contains articles on many of the points above.

Software and code

Policy information about availability of computer code

Data collection	Microarray image analysis : Feature Extraction version 10.7.3.1 (Agilent Technologies), Biomek® NX span-8 laboratory automation workstation: Biomek Software version 4.1 (BECKMAN COULTER)
Data analysis	Genomic mutations were identified by using breseq version 0.30.1 pipeline. For supervised PCA via random forest and hierarchical clustering, we used custom code written in Python 3.6 which is available on GitHub (https://github.com/jiwasawa/evolved-strain-analysis). The stats package in R version 3.2.3. was used for the Mann-Whitney U-test. ImageJ 1.5.2k was used for quantifying single cell area sizes. For all other quantitative analysis, we used custom code written in Python 3.6, relying on scipy 1.1.0, numpy 1.15.4, pandas 0.23.4, and scikit_learn 0.22.1. All details in these scripts that are important to the analysis (e.g. mathematical equations) are documented in the paper.

For manuscripts utilizing custom algorithms or software that are central to the research but not yet described in published literature, software must be made available to editors/reviewers. We strongly encourage code deposition in a community repository (e.g. GitHub). See the Nature Research guidelines for submitting code & software for further information.

Data

Policy information about availability of data

All manuscripts must include a data availability statement. This statement should provide the following information, where applicable:
- Accession codes, unique identifiers, or web links for publicly available datasets
- A list of figures that have associated raw data
- A description of any restrictions on data availability

All microarray data are available in the National Center for Biotechnology Information's Gene Expression Omnibus functional genomics data repository under accession number GSE137348 (https://www.ncbi.nlm.nih.gov/geo/query/acc.cgi?acc=GSE137348). The raw sequence data of genome sequence analyses are available in the DDBJ Sequence Read Archive under the accession number DRA006396. Data supporting the findings of this study are available within the paper and supplementary information files. Source data are provided with this paper.

Field-specific reporting

Please select the one below that is the best fit for your research. If you are not sure, read the appropriate sections before making your selection.

- [x] Life sciences
- [ ] Behavioural & social sciences
- [ ] Ecological, evolutionary & environmental sciences

For a reference copy of the document with all sections, see nature.com/documents/nr-reporting-summary-flat.pdf

Life sciences study design

All studies must disclose on these points even when the disclosure is negative.

Sample size	We did not perform sample-size calculation for statistical robustness. Sample sizes were chosen based on similar published studies.
Data exclusions	All acquired data were used for the statistical analysis.
Replication	For statistical robustness, we controlled the false discovery rate using the Bonferroni correction for detecting pairs of stresses showing cross resistance or collateral sensitivity. Please refer to the methods for more details. We performed six replicates of laboratory evolution. For further phenotypic and genotypic analyses, we selected four evolved strains isolated from each stressor. All attempts at replication were successful. Other experiments were repeated at least two times to ensure reproducibility. Experiments were conducted independently and all atempts at replication were successful.
Randomization	This work did not involve group analyses that require randomization.
Blinding	Randomization is not relevant to this study, because the automated data collection and analysis were not biased by investigators.

Reporting for specific materials, systems and methods

We require information from authors about some types of materials, experimental systems and methods used in many studies. Here, indicate whether each material, system or method listed is relevant to your study. If you are not sure if a list item applies to your research, read the appropriate section before selecting a response.

Materials & experimental systems

n/a	Involved in the study	
x		Antibodies
x		Eukaryotic cell lines
x		Palaeontology
x		Animals and other organisms
x		Human research participants
x		Clinical data

Methods

n/a	Involved in the study	
x		ChIP-seq
x		Flow cytometry
x		MRI-based neuroimaging
